# Integrated ERK‐PKA‐YAP/TAZ‐SHH Signaling Orchestrates Cortical Radial Glia Identity and Lineage Diversification

**DOI:** 10.1002/advs.202513571

**Published:** 2025-11-12

**Authors:** Zhuangzhi Zhang, Zhejun Xu, Tongye Fu, Jialin Li, Feihong Yang, Chuannan Yang, Wenhui Zheng, Zizhuo Sha, Yanjing Gao, Mengge Sun, Zhenmeiyu Li, Jing Ding, Xiaosu Li, Zhengang Yang

**Affiliations:** ^1^ State Key Laboratory of Brain Function and Disorders Ministry of Education Frontiers Center for Brain Science Institutes of Brain Science, and Department of Neurology Zhongshan Hospital Fudan University Shanghai 200032 China

**Keywords:** cortical evolution, cortical gliogenesis, cortical neurogenesis, ERK, PKA, SHH signaling, YAP

## Abstract

The signaling pathways governing cortical neurogenesis and gliogenesis in mice are well‐defined, yet how they integrate to control the lineage progression of cortical radial glia (RGs) remains incompletely understood. Here, using mouse genetic models, it is demonstrated that ERK and PKA signaling cooperate to preserve the neurogenic capacity of cortical RGs by suppressing the gliogenic pathways YAP/TAZ and SHH. Specifically, YAP/TAZ signaling drives cortical RGs toward an ependymal fate, while SHH signaling promotes the generation of tripotential intermediate progenitor cells that produce cortical astrocytes and oligodendrocytes, and olfactory bulb interneurons. Reanalysis of published human cortical scRNA‐seq data further revealed that the functional roles of these signaling pathways are conserved between mouse and human cortical RGs. Furthermore, human cortical outer RGs acquire dominant ERK/PKA signaling through a self‐reinforcing loop that suppresses both YAP and SHH signaling, markedly enhancing self‐renewal and extending neurogenesis. Thus, a tripartite network of ERK/PKA, YAP/TAZ, and SHH whose cross‐repressive logic coordinates neurogenesis with gliogenesis and may underlie evolutionary expansion, providing a framework for understanding cortical development and evolution, is identified.

## Graphical abstract

This study shows that ERK/PKA upregulation in mammalian cortical radial glia (RGs) sustains neurogenesis and suppresses gliogenesis. YAP/TAZ activation promotes ependymal cell formation, while enhanced SHH‐SMO signaling induces astrocyte, oligodendrocytes, and olfactory bulb interneuron generation. In human cortical outer RGs (oRGs), strong ERK/PKA signaling enhances self‐renewal and extends neurogenic period, strictly restricting oRGs to neuronal production.

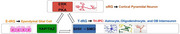

## 1 Introduction

Radial glial (RGs), spanning the entire thickness of the developing cortex, are primary neural stem cells.^[^ ^1^, ^2^, ^3^, ^4^, ^5^, ^6^, ^7^, ^8^ ^]^ On the basis of cell lineage, we define three distinct types of radial glia (RGs) in the developing mouse cortex: neurogenic RGs (N‐RGs), ependymocyte‐generating RGs (E‐RGs), and tripotential intermediate progenitor cell (Tri‐IPC)‐generating RGs (T‐RGs). The early cortical RGs are N‐RGs, generating intermediate progenitor cells (IPCs) exclusively for glutamatergic pyramidal neurons (PyNs), known as PyN‐IPCs.^[^ ^1^, ^2^, ^3^, ^4^, ^5^, ^6^, ^7^, ^8^ ^]^ At the end of cortical neurogenesis, a subpopulation of N‐RGs in the medial cortex transitions into E‐RGs that mainly produce ependymal cells along a medial‐to‐lateral gradient.^[^ ^9^, ^10^, ^11^, ^12^ ^]^ Meanwhile, another N‐RG population transforms into T‐RGs in a lateral‐to‐medial direction, giving rise to cortical Tri‐IPCs that express ASCL1, EGFR, and OLIG1/2.^[^ ^4^, ^13^, ^14^, ^15^, ^16^, ^17^, ^18^ ^]^ Cortical Tri‐IPCs, as their name suggests, exhibit remarkable plasticity and differentiation potential. Indeed, during cortical gliogenesis, Tri‐IPCs give rise sequentially to astrocyte lineage‐restricted IPCs (APCs), oligodendrocyte lineage‐restricted IPCs (OPCs), and IPCs destined for generating cortically derived olfactory bulb interneurons (OBIN‐IPCs). These lineage‐restricted IPCs then divide symmetrically to generate cortical astrocytes, oligodendrocytes, and OBINs, respectively.^[^ ^13^, ^14^, ^15^, ^16^, ^17^, ^18^, ^19^ ^]^ Loss‐of‐function experiments confirm that mouse cortical Tri‐IPCs genuinely possess tripotent differentiation capacity. Notch inhibition restricts cortical Tri‐IPC output to OPCs and OBIN‐IPCs, while APC production is abolished.^[^ ^15^, ^20^ ^]^ *Olig1/2* loss shifts differentiation toward cortical APCs and OBIN‐IPCs at the expense of OPCs.^[^ ^19^, ^21^ ^]^ Conversely, *Gsx1/2* or *Dlx1/2* deficiency permits APC and OPC formation but eliminates OBIN‐IPCs.^[^ ^22^, ^23^, ^24^ ^]^ Clonal analysis using either neurosphere assays or adherent culture conditions reveals the tripotent potential of single neural stem cells and IPCs under in vitro conditions.^[^ ^14^, ^25^ ^]^

After cortical patterning, the default program of cortical RGs is cortical neurogenesis—the production of PyNs. Cortical patterning and neurogenesis both primarily depend on the GLI repressor, which is mediated by cAMP‐protein kinase A (PKA) signaling.^[^ ^26^, ^27^, ^28^ ^]^ cAMP‐PKA signaling is also operational in later corticogenesis, as evidenced by the expression of GPCR genes *Adcyap1r1* and *Adora2b* in cortical RGs. PKA is activated when ADCYAP1R1 binds its ligand ADCYAP1 from PyNs, triggering GNAS (Gsα)‐mediated stimulation of adenylyl cyclase and cAMP production.^[^ ^29^ ^]^ A parallel route is activated by adenosine binding to ADORA2B.^[^ ^30^ ^]^ Downstream targets of cAMP‐PKA‐CREB signaling include *Dio2* and *Cxcl14,^[^* ^31^, ^32^, ^33^, ^34^ ^]^ with the *Dio2* promoter containing a conserved cAMP response element across species.^[^ ^31^, ^32^, ^33^ ^]^ Consistent with this, we confirmed the expression of *Adcyap1r1*, *Adora2b*, *Dio2*, and *Cxcl14* in mouse cortical RGs at later stages (see below), demonstrating functional PKA activity in these cells. During the mouse cortical neurogenic stage, the ablation of either PKA signaling^[^ ^35^ ^]^ or *Gli2/3* ^[^ ^13^ ^]^ in N‐RGs diverts progenitor fate from cortical PyN‐IPCs to Tri‐IPCs. Similarly, enhanced SHH signaling also promotes the production of Tri‐IPCs.^[^ ^13^, ^15^, ^17^, ^35^ ^]^ The Hippo pathway core kinases MST1/2 and LATS1/2 suppress cell growth by phosphorylating YAP/TAZ, inhibiting their nuclear translocation and transcriptional activity. When localized in the nucleus, YAP/TAZ bind TEAD transcription factors to promote cell proliferation and maintain homeostasis.^[^ ^36^, ^37^, ^38^, ^39^, ^40^ ^]^ Previous studies have demonstrated that YAP signaling (YAP/TAZ activity) is essential for the formation of ependymal cells, which are a specialized class of glia.^[^ ^41^, ^42^ ^]^ Thus, although the signaling pathways that promote and/or protect neurogenesis and gliogenesis in mouse cortical RGs are well‐characterized, it remains incompletely understood how these pathways are integrated to govern both the progressive lineage progression of RGs and the spatiotemporal emergence of distinct cell fates.

Extracellular signal‐regulated kinase (ERK) signaling is a master regulator of cell behavior.^[^ ^43^, ^44^ ^]^ This pathway can be activated in cortical RGs through FGFs binding to FGFR1/2/3, which these cells express.^[^ ^17^, ^43^, ^44^, ^45^, ^46^, ^47^ ^]^ Moreover, ERK and PKA signaling can mutually reinforce each other.^[^ ^48^, ^49^, ^50^ ^]^ PKA acts as a strong negative regulator of the SHH‐SMO signaling,^[^ ^39^, ^40^, ^51^, ^52^, ^53^, ^54^ ^]^ and PKA is also widely recognized for its role in inhibiting YAP signaling.^[^ ^36^, ^37^, ^38^, ^39^, ^40^ ^]^ YAP signaling conversely suppresses both cAMP‐PKA and ERK signaling.^[^ ^55^, ^56^ ^]^ Furthermore, YAP signaling inhibits primary cilium assembly,^[^ ^57^ ^]^ which suppress SHH‐SMO signaling. On the other hand, SHH‐SMO signaling represses PKA signaling.^[^ ^40^, ^51^, ^52^, ^54^ ^]^ The SHH‐SMO signaling may additionally suppress ERK signaling through the activation of PP2A‐mediated mechanisms.^[^ ^58^, ^59^ ^]^ It was also suggested that the functional interplay between SHH and YAP signaling in governing cell type specification is mediated by mutual inhibition.^[^ ^60^ ^]^ While the majority of these studies were not performed in cortical RGs, collectively, these findings prompted us to propose that during cortical development, RGs adopt one of three distinct fates, governed by specific signaling pathways: 1) elevated ERK/PKA activity in cortical N‐RGs sustains neurogenesis and suppresses gliogenesis through repression of SHH and YAP signaling; 2) YAP/TAZ activation promotes cortical E‐RG formation and ependymal differentiation by inhibiting both ERK/PKA and SHH signaling; and 3) augmented SHH signaling promotes cortical T‐RG formation and the subsequent generation of Tri‐IPCs, at the expense of ERK/PKA and YAP signaling. To test this hypothesis, we utilized complementary mouse genetic strategies, encompassing both loss‐ and gain‐of‐function experiments.

## 2 Results

### 2.1 YAP Signaling Controls Cortical E‐RG Identity

**Figure 1:**
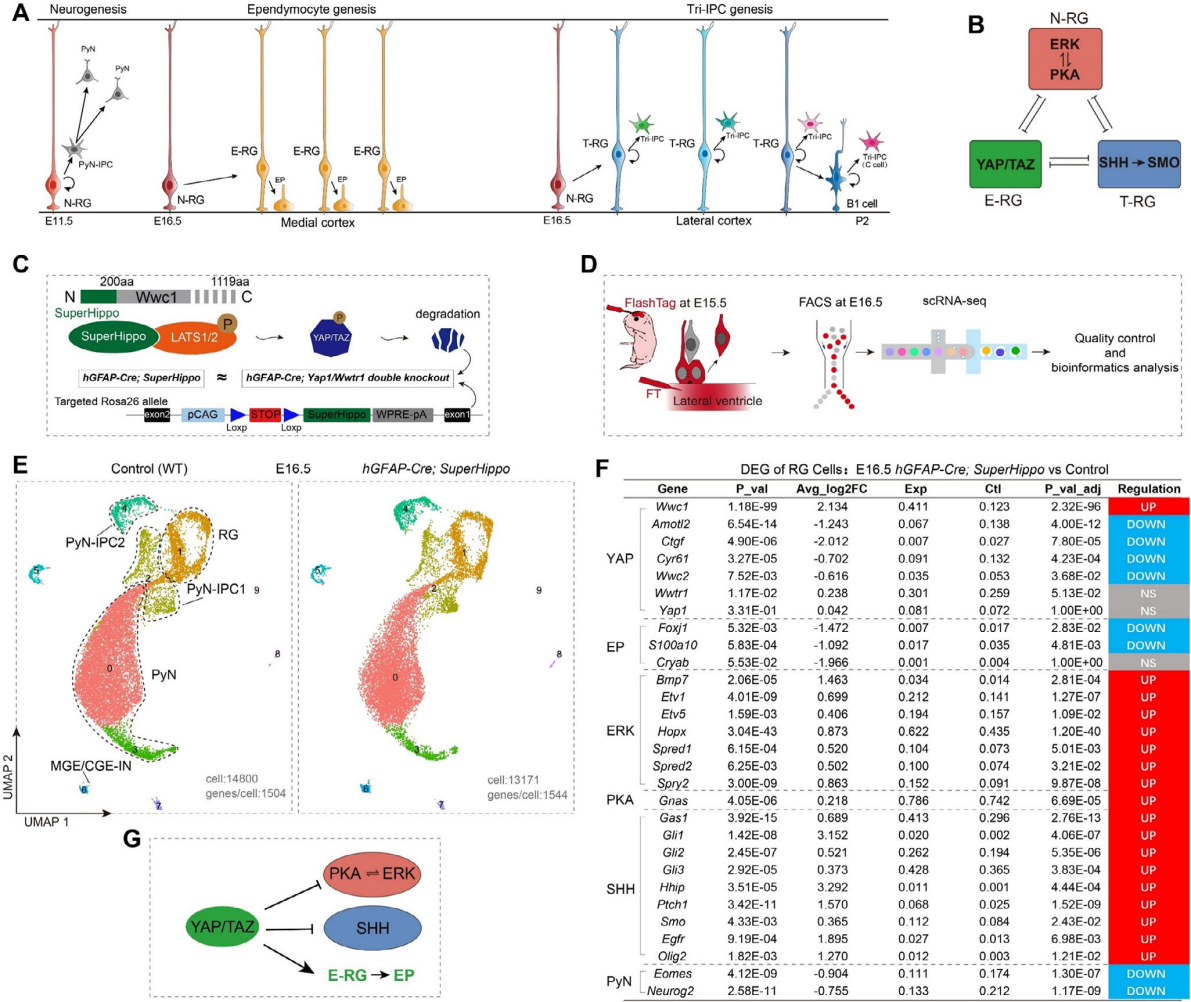
YAP signaling promotes the cortical E‐RG identity. A) The lineage progression of mouse cortical RGs involves sequential transitions. First, N‐RGs constitute the early cortical RG population. Subsequently, around E16.5—coinciding with the end of cortical neurogenesis—two distinct transitions occur: first, N‐RGs in the medial cortex transition into E‐RGs along a medial‐to‐lateral gradient, primarily producing ependymal cells; second, another N‐RG population transforms into T‐RGs in the opposite (lateral‐to‐medial) direction, which then generate cortical Tri‐IPCs. It is possible that cortical T‐RGs transform directly into adult cortical neural stem cells (B1 cells) during postnatal development. B) We propose that a tripartite signaling network—comprising the ERK/PKA, YAP/TAZ, and SHH‐SMO pathways and their cross‐repressive interactions—governs the maintenance of distinct identities in mouse cortical N‐RGs, E‐RGs, and T‐RGs. C) A *SuperHippo* minigene has been engineered to robustly inhibit YAP signaling. D) Strategy for labeling (lateral ventricle injection of FlashTag‐CellTrace Yellow at E15.5) and enriching cortical progenitor cells from two littermate controls (wild type, WT) brains and two *hGFAP‐Cre; SuperHippo* brains at E16.5 and scRNA‐Seq analysis. E) Annotation of cortical cell clusters. F) scRNA‐Seq analysis revealed DEGs in E16.5 cortical RGs (cluster 1 in A) of *hGFAP‐Cre; SuperHippo* mice relatively to WT controls. EP, ependymal cell. G) YAP signaling governs cortical E‐RG identity maintenance and ependymal differentiation by coordinately inhibiting PKA/ERK activity and suppressing SHH singling.

Our hypothesis posits that a tripartite signaling network—comprising the ERK/PKA, YAP/TAZ, and SHH pathways and their cross‐repressive interactions—regulates cortical lineage progression, cortical neurogenesis, ependymal gliogenesis, and macroglial (Tri‐IPC) genesis in cortical RGs (**Figure** **1**A,B). To investigate the role of YAP signaling in cortical N‐RGs, we crossed mice harboring a *WWC*‐derived floxed minigene (referred to as *SuperHippo*) with *hGFAP‐Cre* mice. *SuperHippo* specifically inhibits YAP signaling, mimicking *Yap1* and *Wwtr1* (*Taz*) double knockout mice (Figure 1C).^[^ ^61^ ^]^ To label E15.5 progenitors, we injected FlashTag into the mouse lateral ventricle. At E16.5, we isolated FlashTag‐labeled cortical cells for single‐cell RNA sequencing (scRNA‐Seq) analysis (Figure 1D). Blocking YAP signaling reduced expression of *Cyr61* (*Ccn1*)*, Ctgf* (*Ccn2*)*, Amotl2*, and *Wwc2* in cortical RGs, and eliminated expression of *Cryab* and *Foxj1* (early ependymal cell markers) (Figure 1E,F). On the other hand, ERK signaling response genes (e.g., *Etv1, Etv5, Spred1/*2, *Spry2, Bmp7*, and *Hopx*) were upregulated (Figure 1F). *Gnas*, a signature PKA signaling gene, was also increased, suggesting that a subpopulation of cortical RGs in *hGFAP‐Cre; SuperHippo* mice at E16.5 exhibit higher PKA signaling (Figure 1F). The *hGFAP‐Cre; SuperHippo* mutant had increased SHH‐SMO signaling based in upregulation of *Gas1, Gli1, Gli2, Gli3, Hhip, Ptch1*, *Smo, Egfr, and Olig2* in cortical RGs (Figure 1F). ERK signaling enhances self‐renewal of cortical RGs,^[^ ^17^, ^62^, ^63^, ^64^ ^]^ while SHH‐SMO signaling promotes the generation of Tri‐IPCs from cortical RGs.^[^ ^13^, ^15^ ^]^ Correspondingly, expression of PyN‐IPC marker genes *Eomes* (*Tbr2*) and *Neurog2* was downregulated in the mutant cortical RGs (Figure 1F).

**Figure 2:**
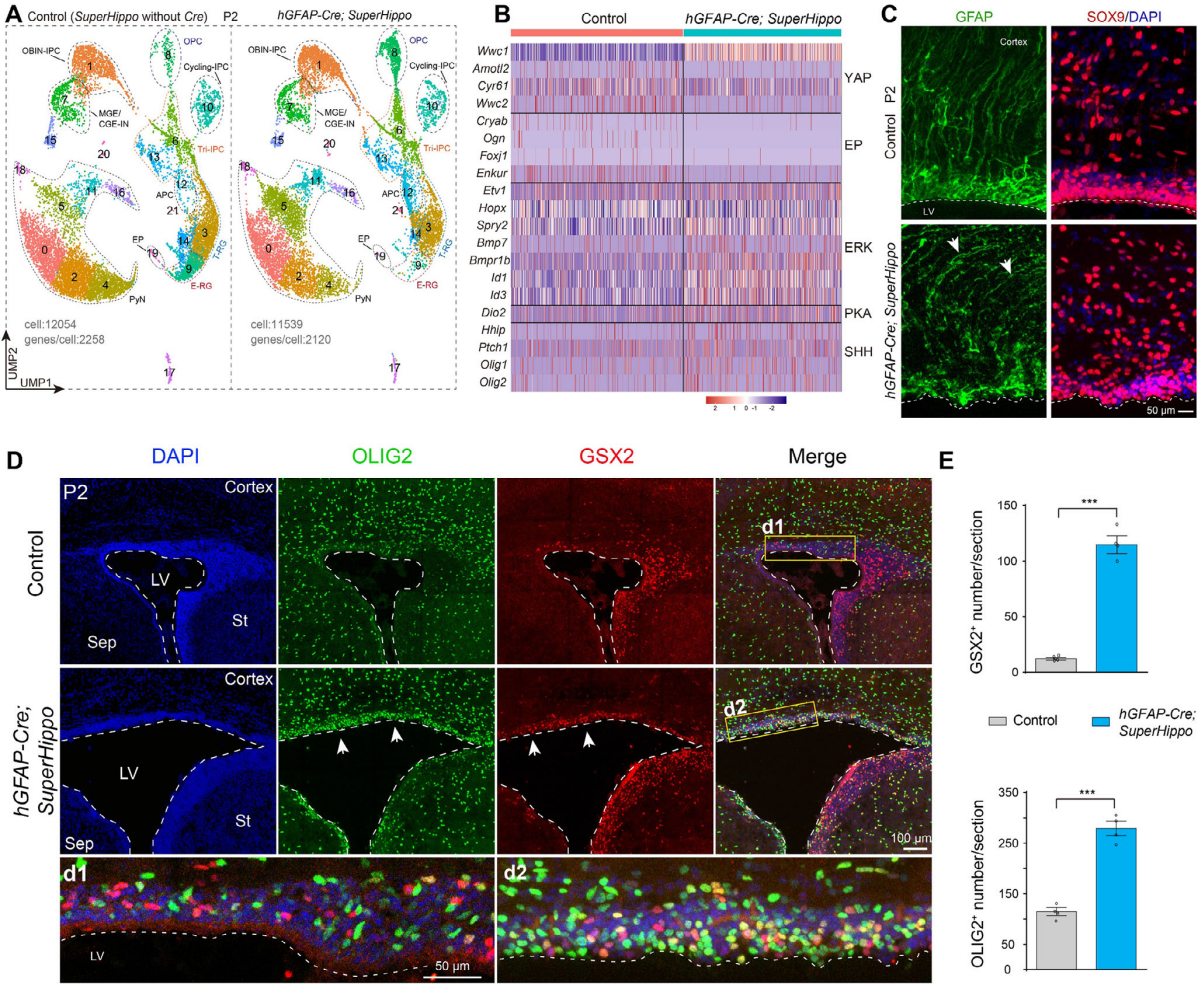
YAP signaling serves as a master regulator of cortical ependymal cell production from RGs. A) Annotation of cortical cell clusters in P2 control (*SuperHippo* heterozygotes without *hGFAP‐Cre*) and *hGFAP‐Cre; SuperHippo* mice. B) Heatmap illustrating DEGs in cortical T‐RGs (cluster 3 in A). C) Immunostaining reveals disruption of the cortical ventricular surface and the loss of apical SOX9‐ and GFAP‐expressing immature ependymal cells in the P2 *hGFAP‐Cre; SuperHippo* cortex. LV, lateral ventricle. D,E) Immunostaining reveals disruption of the cortical ventricular surface and increase in OLIG2‐ and GSX2‐expressing progenitors in the cortical VZ/SVZ of *hGFAP‐Cre; SuperHippo* mice at P2 (*n* = 4 per group; mean ± SEM; ***P <0.001; unpaired two‐tailed Student's *t*‐test).

By P2, cortical E‐RGs and their ependymal cell progeny were absent in *hGFAP‐Cre; SuperHippo* mice (**Figure** **2**A,B; and Figure S1A,B, Supporting Information), consistent with prior reports demonstrating the essential role of YAP signaling in ependymal cell development.^[^ ^41^, ^42^ ^]^ Immunohistochemical analysis demonstrated an increase in Tri‐IPCs (OLIG2‐ and GSX2‐expressing cells) and the loss of ependymal cells (Figure 2C–E). Although cortical E‐RGs and ependymal cells are lost in *hGFAP‐Cre; SuperHippo* mice, cortical T‐RGs were preserved but still showed downregulation of YAP‐signaling genes, and the expression of *Cryab, Foxj1*, and *Ogn* (ependymal cell markers), was abolished (Figure 2A–C; and Figure S1B, Supporting Information). In contrast, ERK, PKA, and SHH signaling were upregulated (Figure 2B; and Figure S1, Supporting Information). All of these findings were further confirmed via scRNA‐Seq and immunohistochemical analysis of *Emx1‐Cre; SuperHippo* mice (Figure S2A–E, Supporting Information). Moreover, the phenotypes observed at E16.5 were more pronounced in *Emx1‐Cre; SuperHippo* than in *hGFAP‐Cre; SuperHippo* mice (Figure 1F; and Figure S2B, Supporting Information). This difference can be attributed to the earlier onset of Cre activity. Specifically, *Emx1‐Cre* is active in cortical neuroepithelial cells starting at E10.5,^[^ ^65^ ^]^ whereas *hGFAP‐Cre* begins to drive expression in cortical RGs around E13.0.^[^ ^66^ ^]^

We also reanalyzed bulk RNA‐Seq and ChIP‐Seq data from mice expressing the YAP1‐MAMLD1 (YAP‐M) fusion protein, which is implicated in supratentorial ependymoma.^[^ ^67^ ^]^ The gene expression profiles demonstrated distinct patterns between cells from the SVZ of wild‐type mice (*n* = 3) and tumor cells derived from ≈P20 mice transfected with YAP‐M at E14.5 (*n* = 4) (Figure S3A, Supporting Information). The YAP‐M fusion oncogene significantly upregulated expression of YAP signaling components and early ependymal cell markers (Figure S3A, Supporting Information). Conversely, YAP‐M oncogene exhibited a strong suppressive effect on expression key signaling pathway genes, including ERK, PKA, and SHH cascades, in addition to genes characteristic of cortical progenitor populations, such as Tri‐IPCs, APCs, OPCs, and OBIN‐IPCs (Figure S3A, Supporting Information). This is consistent with observations that human YAP‐M‐driven tumor cells lack classical glial progenitor markers.^[^ ^67^, ^68^ ^]^ YAP1 and WWTR1 are transcriptional coactivators. Reanalysis of ChIP‐Seq and Cut&Run data for YAP1 and H3K27ac in both YAP‐M‐induced tumor cells and YAP1‐expressing mouse neural stem cells identified multiple YAP‐M target genes, characterized by distinct YAP‐M binding sites, mainly in the promoter region.^[^ ^69^ ^]^ These direct target genes included *Foxj1, Cryab, Anxa2, S100a11*, and *Ogn*, among others (Figure S3B,C, Supporting Information). Taken together, these loss‐ and gain‐of‐function analyses demonstrate that YAP signaling promotes cortical E‐RG identity during cortical development and suppresses ERK, PKA, and SHH signaling (Figure 1G).

### 2.2 SHH‐SMO Drives the Establishment of Cortical T‐RG Identity

**Figure 3:**
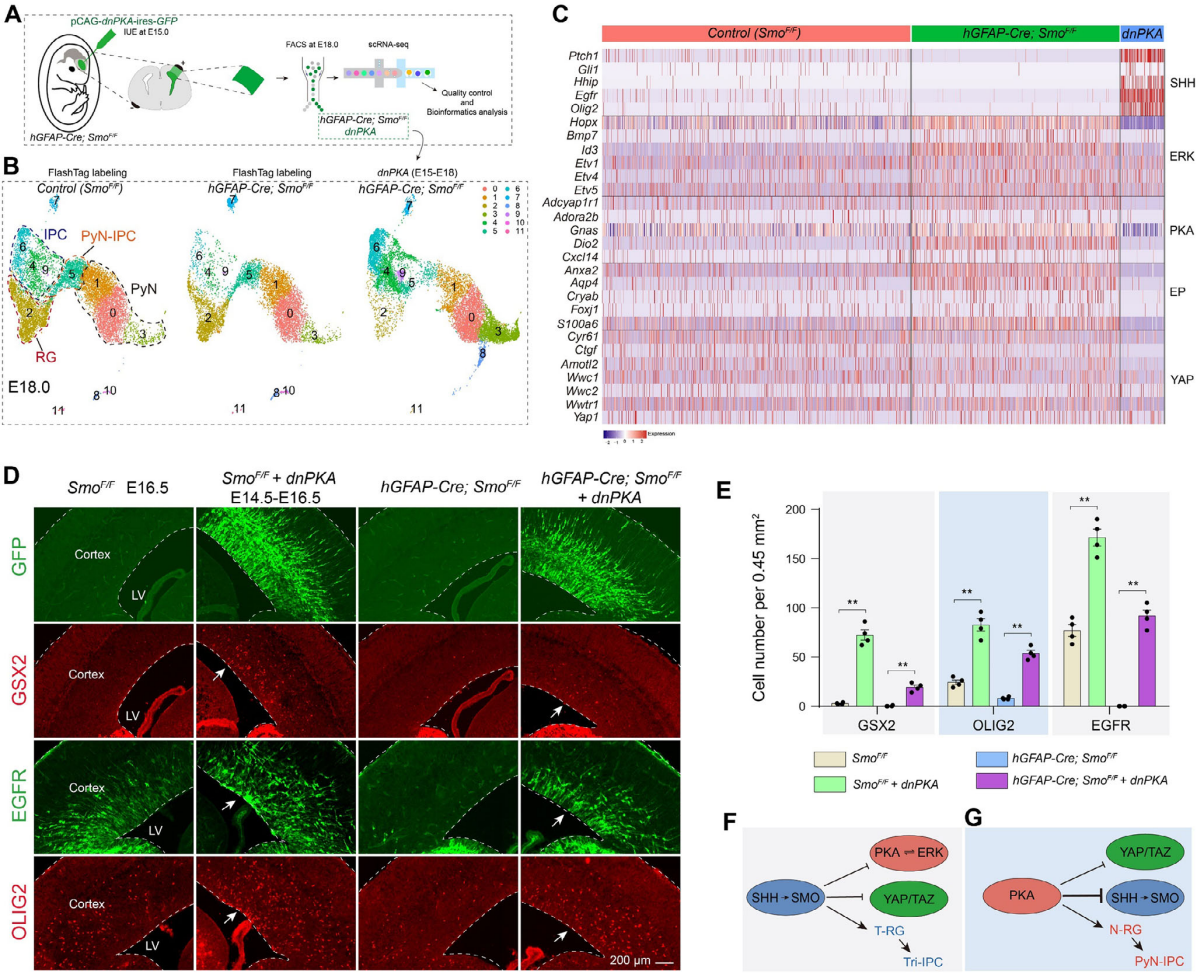
The functional interplay between SHH and PKA signaling in the cortical RGs. A) Scheme for overexpressing *pCAG‐dnPKA‐GFP* plasmids into the cortical VZ of *Smo^F/F^* control and *hGFAP‐Cre; Smo^F/F^* mouse embryos at E15 by IUE, and scRNA‐Seq of GFP‐expressing cells at E18.0. B) Annotation of cortical cell clusters. C) Heatmap illustrating gene expression in cortical RGs (cluster 2 in B) with IUE of *dnPKA*. D,E) Immunostaining reveals a marked expansion of GSX2‐, EGFR‐, and OLIG2‐positive progenitors in the cortical VZ/SVZ following IUE of *dnPKA* (PKA signaling completely ablation) (*n* = 4 per group; mean ± SEM; **P <0.01; one‐way ANOVA and Tukey's post‐hoc test). F) SHH‐SMO signaling promotes the identity of T‐RGs. G) PKA signaling maintains cortical N‐RG identity by strongly inhibiting SHH signaling and moderately suppressing YAP signaling.

In all studied animals, a primary function of activated SMO is to counteract the inhibitory effects of PKA on GLI transcription factors.^[^ ^51^ ^]^ To investigate the *Smo* function in cortical RGs, we reanalyzed our previous scRNA‐Seq data of cortical cells in *hGFAP‐Cre; Smo^F/F^* mice E18.0.^[^ ^35^ ^]^ In the absence of *Smo*, cortical RG expression of *Gli1, Hhip, Ptch1, Egfr*, and *Olig2* was nearly undetectable (**Figure** **3**A–C). In contrast, signature genes associated with ERK signaling (*Hopx, Bmp7, Etv5*, etc.), PKA signaling (*Adcyap1r1, Adora2b, Gnas, Dio2*, and *Cxcl14*), and early ependymal cell markers (*Anxa2, Aqp4, Cryab, Foxj1*, and *S100a6*) exhibited significant upregulation (Figure 3C). Notably, the observed upregulation of YAP signaling‐responsive genes was not pronounced (Figure 3C). It is likely that this modest change in expression at E18 is due to the potent inhibitory effect of PKA signaling on YAP activity. Additionally, the intensity of ventral‐derived SHH‐SMO signaling exhibits an inherently low level prenatally, which increases significantly after birth in mice.^[^ ^70^ ^]^ To further validate these findings, we conducted additional scRNA‐Seq analysis of cortical cells isolated from *hGFAP‐Cre; Smo^F/F^* mice and their *Smo^F/^* ^F^ littermate controls at P2. Notably, both E‐RGs and T‐RGs exhibited upregulation of ERK, PKA, and YAP signaling pathways (Figure S4A–C, Supporting Information). Taken together, these findings indicate that SHH‐SMO signaling promotes and maintains mouse cortical T‐RG identity and facilitates their differentiation into Tri‐IPCs by suppressing ERK, PKA, and YAP signaling (Figure 3F).

### 2.3 PKA Protects Cortical N‐RG Identity

The reciprocal regulatory relationship between SHH signaling and PKA activity is well characterized: elevated PKA activity suppresses SHH signaling, while reduced PKA activity potentiates SHH pathway activation.^[^ ^51^, ^52^, ^54^ ^]^ To further investigate the functional role of PKA in cortical RGs under conditions of *Smo* deficiency, we next performed a series of genetic analyses. At E15, we conducted IUE to ectopically express a dominant‐negative form of PKA (d*nPKA‐GFP*) in the mouse cortex. By E18, cells expressing *dnPKA‐GFP* were isolated and analyzed by scRNA‐Seq. Comparative analysis with FlashTag labeled cells from control (*Smo^F/F^*) and *hGFAP‐Cre; Smo^F/F^* E18 mice without *dnPKA*‐IUE revealed the following findings in the cortical RGs of *hGFAP‐Cre; Smo^F/F^* mice expressing *dnPKA* (Figure 3A–C; and Figure S5A,B, Supporting Information): 1) complete ablation of PKA signaling; 2) significant upregulation of SHH pathway genes; 3) marked downregulation of ERK signaling; 4) suppression of YAP signaling; 5) blockade of early ependymal cell marker genes. Thus, the complete loss of PKA in cortical RGs directly leads to a significant increase in SHH signaling (GLI activators), even without *Smo* function, which subsequently represses both ERK and YAP signaling. This result closely mirrors the phenotypes observed in our prior studies involving the overexpression of *Shh‐N, Gli1, Gli2 activator*, or *SmoM2* in cortical RGs.^[^ ^13^, ^15^, ^17^, ^35^ ^]^ Our results further solidify the role of PKA as the principal negative regulator of SHH signaling,^[^ ^51^, ^52^, ^54^ ^]^ in addition to its role as a suppressor of YAP signaling.^[^ ^36^, ^40^ ^]^ Immunostaining analysis of cortical sections 48‐h after *dnPKA* IUE revealed upregulated expression of EGFR, OLIG2, and GSX2 in the cortex of control and *hGFAP‐Cre; Smo^F/F^* mice (Figure 3D,E). This highlights the pivotal role of PKA signaling in N‐RGs (neurogenic RGs) in protecting PyN genesis while simultaneously suppressing gliogenesis, including the production of ependymal cells, astrocytes, oligodendrocytes, and OBINs (Figure 3G).

Therefore, genetic perturbations, including loss of SHH function (*hGFAP‐Cre; Smo^F/F^*), gain of SHH function, and loss of PKA function (both via *dnPKA* overexpression), further support our model. This model posits that a tripartite signaling network, centered on cross‐repressive interactions among the ERK/PKA, YAP, and SHH pathways, regulates mammalian cortical neurogenesis, ependymal gliogenesis, and macroglial (Tri‐IPC) genesis (Figure 3F,G).

### 2.4 ERK Safeguards both Cortical N‐RG and T‐RG Identity

**Figure 4:**
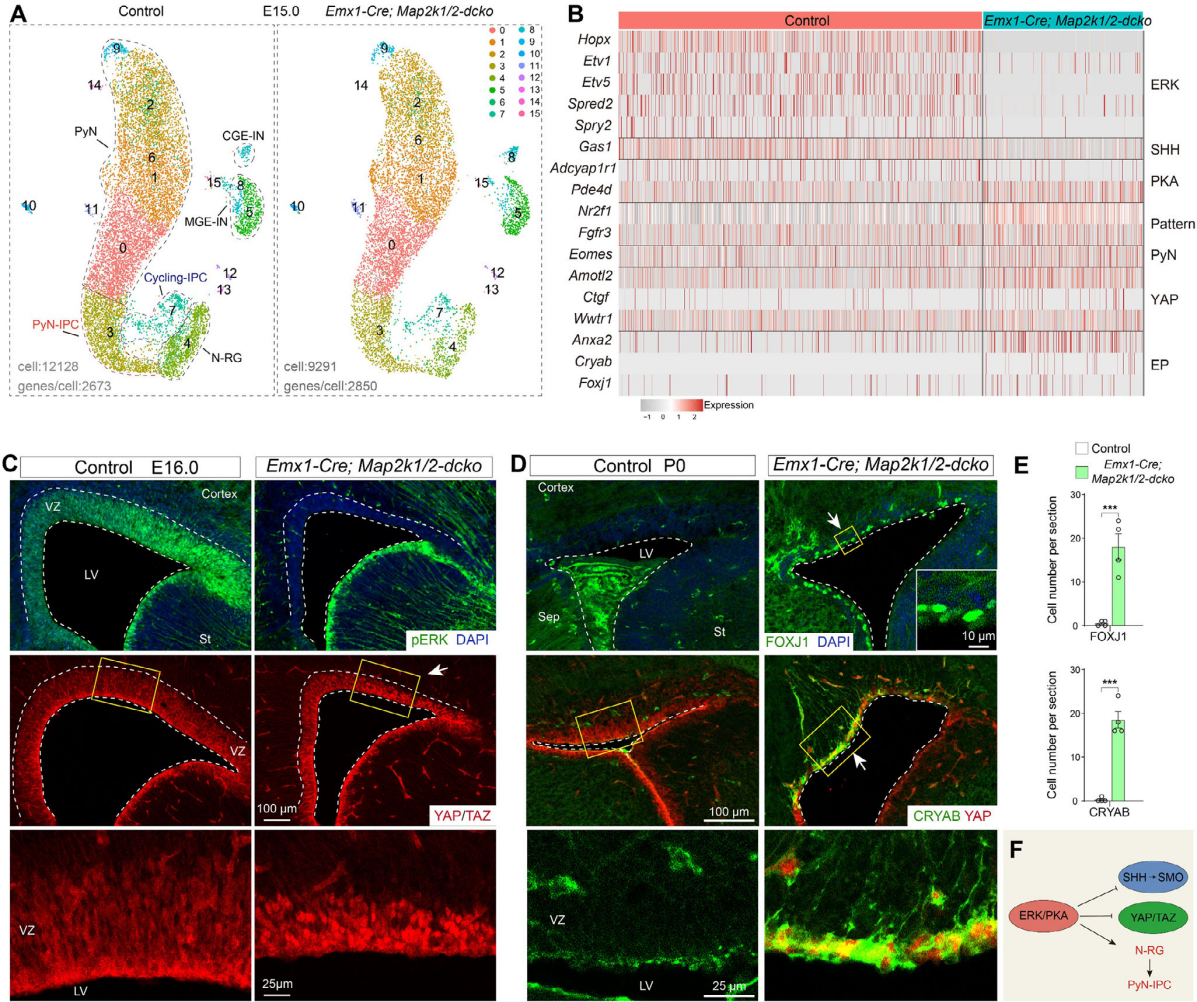
ERK signaling enhances PKA signaling and suppresses YAP signaling. A) UMAP showing annotated cell clusters based on scRNA‐Seq of E15.0 control and *Emx1‐cre; Map2k1/2‐dcko* cortical cells. N‐RG: neurogenic RG. B) Heatmap depicting DEGs across selected ERK, SHH, PKA, and YAP signaling response genes, along with cortical regional patterning markers and E‐RG genes within cortical N‐RGs. C) pERK (phosphorylated ERK) staining is lost, and nuclear localization of YAP/TAZ proteins is increased specifically in cortical RGs of *Emx1‐Cre; Map2k1/2‐dcko* mice at E16.0. D,E) FOXJ1‐ and CRYAB‐expressing cells are observed in the VZ of *Emx1‐Cre; Map2k1/2‐dcko* mice, whereas their expression is undetectable in controls at P0 (*n* = 4 per group; mean ± SEM; ***P <0.001; unpaired two‐tailed Student's *t*‐test). Sep, septum; St, striatum. F) ERK/PKA signaling maintains the neurogenic program of cortical N‐RGs by repressing gliogenic YAP and SHH signaling.

ERK signaling regulates multiple steps in cortical development.^[^ ^15^, ^43^, ^44^, ^45^, ^46^, ^47^ ^]^ To examine the function of ERK signaling, we used both *Emx1‐Cre* and *hGFAP‐Cre* lines to conditionally delete *Map2k1* and *Map2k2* (core downstream components of ERK; *Map2k1/2‐dcko*) at progressively later stages.^[^ ^15^, ^17^ ^]^ scRNA‐Seq analysis of E15.0 *Emx1‐Cre*; *Map2k1/2‐dcko* cortices revealed that SHH coreceptor *Gas1* expression was downregulated (**Figure** **4**A,B), and expression of ERK signaling downstream target genes was completely lost in N‐RGs (Figure 4B). This results in a significant decrease in the population of cortical N‐RGs, concurrent with elevated *Eomes* expression (Figure 4B). These cellular alterations were associated with pronounced microcephaly, as evidenced by a substantial reduction in overall brain size (Figure S6A–C, Supporting Information), again demonstrating that ERK signaling in cortical N‐RGs promotes self‐renewal through enhanced cell proliferation while simultaneously inhibiting the generation of PyN‐IPCs.^[^ ^17^, ^62^, ^63^, ^64^ ^]^ The upregulation of *Nr2f1* and *Fgfr3* expression in N‐RGs confirmed that ERK signaling is essential for promoting the rostral cortical identity (Figure 4B).^[^ ^17^, ^45^ ^]^ Most importantly, we observed a subpopulation of cortical N‐RGs exhibited *Adcyap1r1* expression, which was markedly downregulated in *Emx1‐Cre; Map2k1/2‐dcko* mice (Figure 4B). Conversely, there was upregulation of *Pde4d* expression in N‐RGs (Figure 4B). ERK‐mediated phosphorylation of PDE4D3 at Ser579 inhibits PDE4 enzymatic activity, thereby potentiating cAMP‐PKA signaling pathways.^[^ ^50^ ^]^ Consequently, ERK deficiency initiates a signaling cascade characterized by attenuated PKA activity, leading to YAP signaling activation (upregulation of *Ctgf*, *Amotl2*, and *Wwtr1*) and elevated expression of early ependymal cell marker genes (*Anxa2*, *Cryab*, and *Foxj1*) in cortical RGs of *Emx1‐Cre; Map2k1/2‐dcko* mice at E15.0 (Figure 4B).

**Figure 5:**
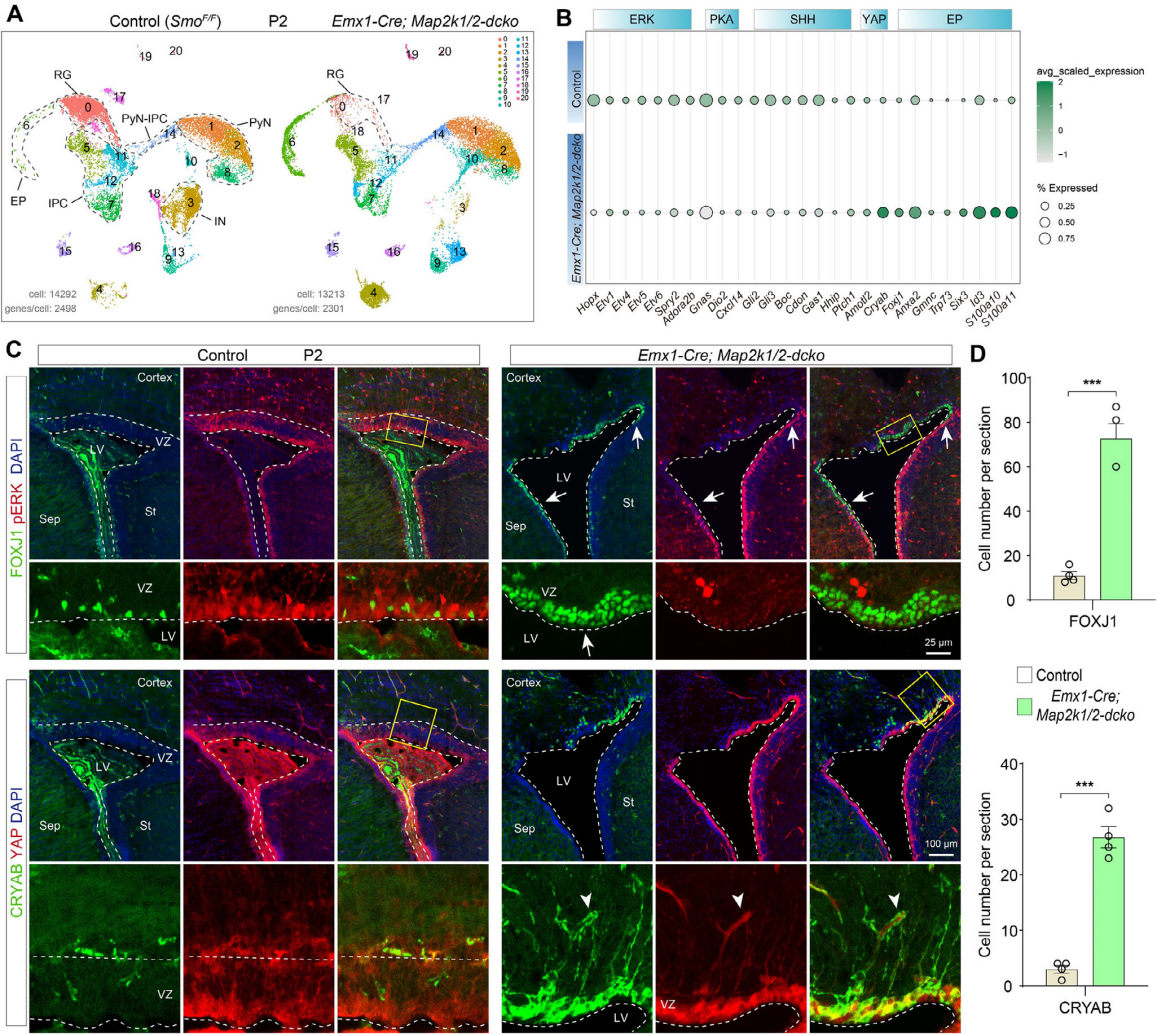
Loss of ERK signaling leads to downregulated PKA and SHH signaling, and increased YAP signaling. A) UMAP showing annotated cell clusters based on scRNA‐Seq analysis of P2 control and *Emx1‐cre; Map2k1/2‐dcko* cortical cells. B) Bubble plot displaying DEGs among selected ERK, PKA, SHH, and YAP signaling response genes, as well as early cortical ependymal cell markers, in cortical RGs (cluster 0 in A) of *Emx1‐Cre; Map2k1/2‐dcko* mice compared controls at P2. C,D) FOXJ1‐ and CRYAB‐expressing cells are significantly increased in the cortical VZ of *Emx1‐Cre; Map2k1/2‐dcko* mice, compared to the few FOXJ1‐ and CRYAB‐expressing cells observed in control mice at P2 (*n* = 4 per group; mean ± SEM; ***P <0.001; unpaired two‐tailed Student's *t*‐test). Note that, in blood vessels, endothelial cells and vascular smooth muscle cells also expressed CRYAB and YAP/TAZ (arrowheads).

To further investigate the conserved functions of ERK signaling, we conducted a comprehensive reanalysis of our scRNA‐Seq datasets from both *Emx1‐Cre; Map2k1/2‐dcko* and *hGFAP‐Cre; Map2k1/2‐dcko* mice at P2.^[^ ^15^ ^]^ While *Emx1‐Cre; Map2k1/2‐dcko* mice displayed a substantial depletion of cortical RGs (**Figure** **5**A,B), both mutants shared RGs defects, including: 1) loss of ERK signaling; 2) reduction of PKA signaling; 3) attenuation of SHH‐SMO signaling; 4) failure to generate Tri‐IPCs; 5) activation of YAP signaling; 6) upregulation of early ependymal cell marker gene expression; and (7) increased production of ependymal cell populations (Figure 5A,B; and Figure S7A–C, Supporting Information). Our immunohistochemistry analyses yielded consistent results: a significant increase in YAP/TAZ nuclear localization within the cortical VZ at E16.0 (Figure 4C), followed by elevated numbers of CRYAB‐ and FOXJ1‐expressing cells at P0 and P2 in *Emx1‐Cre; Map2k1/2‐dcko* mice compared to control littermates (Figures 4D,E, and 5C,D). We next employed a gain‐of‐function approach using *Emx1‐Cre; Mek1DD* mice, which specifically enhances ERK signaling in cortical RGs starting from E10.5. This resulted in a marked expansion of the cortical RG population while simultaneously inhibiting neuronal differentiation, ultimately leading to pronounced macrocephaly (Figure S6D–H, Supporting Information).

In *Emx1‐Cre; Map2k1/2‐dcko* mice, the degradation of pERK downregulates ERK and PKA signaling. While basal PKA activity potently inhibits ventrally‐derived SHH‐SMO signaling, it is insufficient to counteract local YAP activation, which occurs earlier than SHH‐SMO signaling in cortical RGs. Indeed, low‐level YAP/TAZ activity, activated by noncanonical WNT signaling along the cortical midline, was detected as early as E10.5 in cortical RGs.^[^ ^41^, ^71^, ^72^, ^73^ ^]^ This early patterned expression establishes a foundation for the medial‐high to lateral‐low gradient of YAP signaling observed in later stages. This temporal signaling hierarchy (elevated YAP signaling) directs cortical N‐RGs to initially produce E‐RGs, facilitating ependymal cell formation while inhibiting SHH signaling. Consequently, in the *Map2k1/2‐dcko*, there is a block of N‐RGs to differentiate into T‐RGs and their Tri‐IPC progeny.^[^ ^15^ ^]^ However, experimental enhancement of SHH signaling in cortical RGs of *Emx1‐Cre; Map2k1/2‐dcko* mice, which exhibit repressed YAP signaling, rescued Tri‐IPC generation.^[^ ^15^ ^]^ In summary, during mouse cortical neurogenesis, ERK/PKA signaling cooperate to suppress gliogenic YAP/TAZ and SHH signaling, thereby maintaining RG neurogenic potential (Figure 4F). During cortical gliogenesis, however, ERK/PKA signaling in the lateral cortex acts to suppress YAP/TAZ activity, thereby ensuring the generation of cortical Tri‐IPCs.^[^ ^15^ ^]^ This protective effect arises because SHH‐SMO signaling more readily inhibits PKA than it does YAP signaling.^[^ ^39^, ^40^, ^51^, ^52^, ^53^, ^54^ ^]^

### 2.5 Molecular Identity of Human Cortical oRGs, E‐tRGs, and T‐tRGs

**Figure 6:**
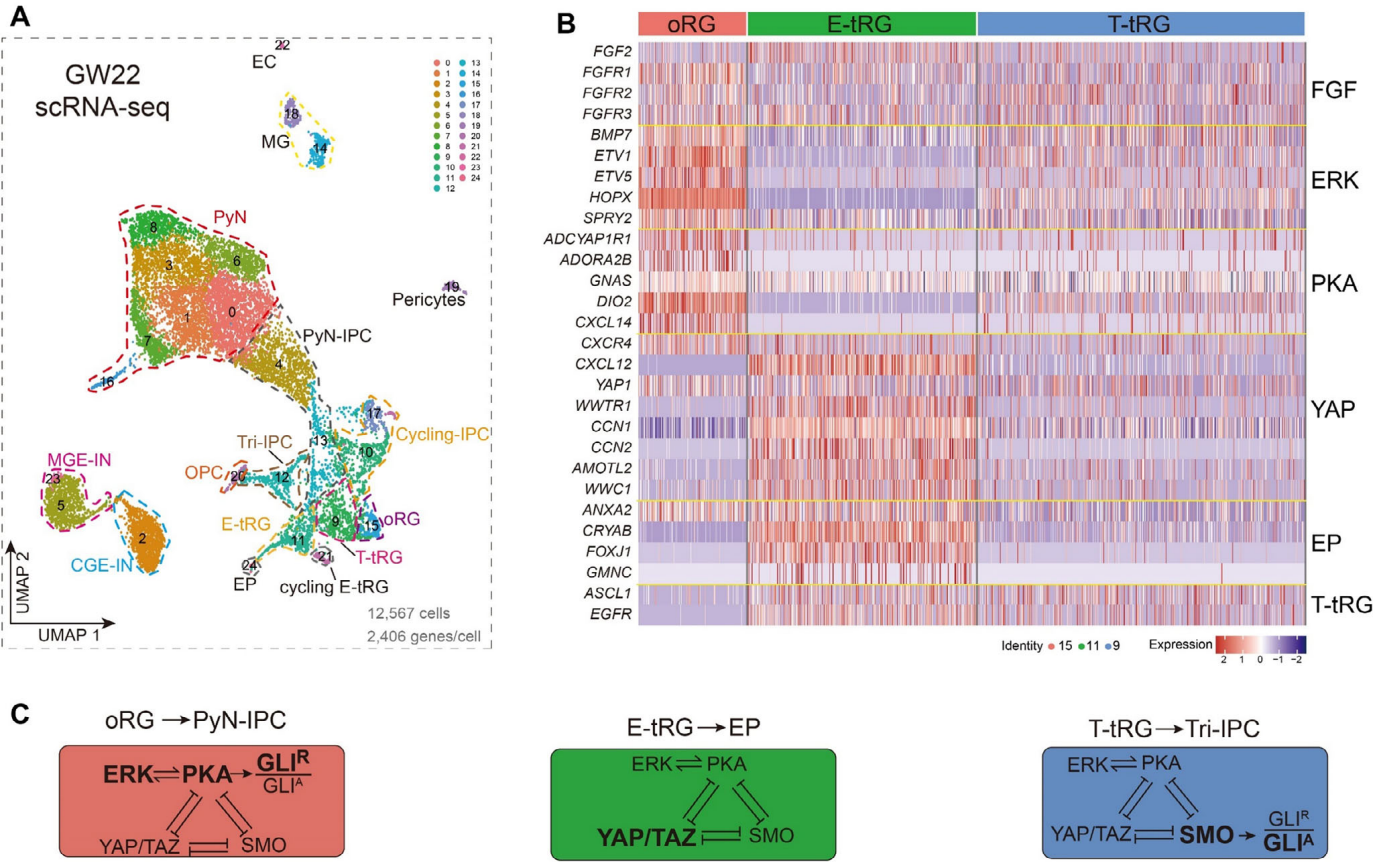
The molecular signatures of human cortical oRGs, E‐tRGs, and T‐tRGs. A) scRNA‐Seq data derived from human cortical tissue at GW22. UMAP visualization with cells colored by cluster. B) Heatmap illustrating differentially expressed genes (DEGs) among human cortical oRGs, E‐tRGs, and T‐tRGs. C) The schematic illustrates distinct signaling and fate potentials: oRGs displayed elevated ERK/PKA signaling and generated PyN‐IPCs; E‐tRGs displayed enhanced YAP signaling and generated ependymal cells; following elevated SHH‐SMO signaling, T‐tRGs displayed suppressed PKA signaling—reflected in lower *DIO2* expression—and began expressing markers like *ASCL1* and *EGFR*, a process that resulted in the production of cortical Tri‐IPCs. GLI^R^, GLI repressor form; GLI^A^, GLI activator form.

During human corticogenesis, full‐span radial glial cells (fRGs) persist from gestational week (GW) 8 through GW16. Around GW16, a large number of fRGs give rise to the truncated radial glia (tRGs) in the ventricular zone (VZ) and the outer radial glia (oRGs, also known as basal RGs) in the outer subventricular zone (outer SVZ, OSVZ).^[^ ^18^, ^35^, ^74^, ^75^, ^76^, ^77^, ^78^, ^79^ ^]^ Human cortical fRGs are neurogenic, mainly generating PyN‐IPCs for deep layer PyNs. Previous studies have provided evidence that human cortical oRGs, which are rare in mice, are also neurogenic, mainly generating PyN‐IPCs for upper layer PyNs.^[^ ^4^, ^15^, ^17^, ^18^, ^35^, ^74^ ^]^ Similar to mouse cortical RGs, human tRGs exhibit three distinct subtypes, referred to as N‐tRGs, E‐tRGs, and T‐tRGs. After human N‐tRGs are generated from fRGs at GW16, they start to express EGFR and ASCL1, and initially generating PyN‐IPCs for upper layer PyNs (i.e., at GW16‐GW17).^[^ ^14^, ^18^, ^80^ ^]^ Subsequently, N‐tRGs undergo a neurogenesis to gliogenesis transition. At approximately GW18, a subset of medial cortical N‐tRGs transitions into E‐tRGs, which subsequently generate the cortical ependymal cells.^[^ ^11^, ^14^, ^18^, ^81^ ^]^ In parallel, another population of lateral cortical N‐tRGs transits into T‐tRGs, giving rise to Tri‐IPCs.^[^ ^14^, ^15^, ^18^, ^35^, ^80^ ^]^ These Tri‐IPCs give rise to human cortical APCs, OPCs, and cortically derived OBIN‐IPCs.^[^ ^4^, ^14^, ^16^, ^18^ ^]^ Therefore, human cortical development exhibits a slower, more refined progression, offering a unique model to investigate the molecular mechanisms underlying neurogenesis, gliogenesis, and evolutionary expansion. To further identify the molecular features of human cortical oRGs, E‐tRGs, and T‐tRGs, we reanalyzed published scRNA‐Seq data using the human GW22 cortical tissue.^[^ ^82^ ^]^ The heatmap presents a comprehensive profile of 28 genes (**Figure** **6**A,B), illustrating the molecular identity and potential cell lineage relationships among human cortical oRGs, E‐tRGs, and T‐tRGs (Figure 6C). Our analysis indicates that multiple pathways likely converge to co‐suppress SHH signaling and YAP/TAZ activity in human cortical oRGs.

While *FGF2* and *FGFR1/2/3* are expressed at comparable levels in human cortical oRGs, E‐tRGs, and T‐tRGs at GW22, ERK signaling intensity varies, being strongest in oRGs, weakest in E‐tRGs, and intermediate in T‐tRGs (Figure 6B). This conclusion stems from the strikingly high expression of ERK signaling response genes, including *BMP7, ETV1, ETV5, HOPX*, and *SPRY*2 (Figure 6B; and Figure S8A,B, Supporting Information).^[^ ^17^, ^35^ ^]^ Moreover, we observed that cAMP‐PKA signaling activity exhibits a pattern similar to that of ERK signaling across RGs. For example, *ADCYAP1R1*, *ADORA2B, GNAS, DIO2*, and *CXCL14* are expressed much higher in oRGs than E‐tRGs and T‐tRGs (Figure 6B; and Figure S8B, Supporting Information), providing evidence that oRGs have high PKA activity. PKA‐mediated phosphorylation of FGF2 increases its binding affinity up to eightfold and potentiates ERK activity.^[^ ^83^ ^]^ Moreover, ERK and PKA signaling can mutually reinforce each other,^[^ ^48^, ^49^, ^50^ ^]^ demonstrating that heightened ERK and PKA activity represents a defining feature of human cortical oRGs.

YAP signaling signature genes are highly expressed in human cortical E‐tRGs, including, *CCN1 (CYR61), CCN2 (CTGF), AMOTL2*, *WWC1/2*, and *WWTR1* (TAZ) (Figure 6B; and Figure S8C, Supporting Information). Consequently, markers for early ependymal cells, including *CRYAB, FOXJ1*, *ANXA2*, and *GMNC*, are also expressed in E‐tRGs (Figure 6B; and Figure S8C, Supporting Information). Furthermore, *CXCL12/CXCR* signaling is upregulated in E‐tRGs compared to oRGs and T‐tRGs (Figure 6B; and Figure S8C, Supporting Information), which represses cAMP‐PKA signaling.^[^ ^40^, ^84^, ^85^ ^]^ Indeed, PKA and ERK signaling are extremely low in E‐tRGs (Figure 6B). Collectively, this analysis provides evidence that YAP signaling, by repression of PKA, ERK, and SHH signaling, is a key feature of human cortical E‐tRGs.

At GW22, T‐tRGs exhibit low to moderate levels of ERK, PKA (*DIO2* expression), YAP, and SHH signaling (Figure 6B; and Figure S8A–D, Supporting Information), indicating the convergence of multiple signaling pathways. Indeed, *EGFR* and *ASCL1*, which are activated by ERK, SHH, and/or YAP signaling, are expressed by a subpopulation of T‐tRGs.^[^ ^15^, ^16^, ^18^, ^35^, ^67^, ^80^ ^]^ These T‐tRGs give rise to Tri‐IPCs that subsequently generate APCs, OPCs, and OBIN‐IPCs.^[^ ^4^, ^14^, ^15^, ^18^, ^35^ ^]^ However, at GW22, we identified APCs and OPCs, but not OBIN‐IPCs, derived from Tri‐IPCs (Figure S9A–F, Supporting Information). With increased SHH signaling in the cortical VZ at GW23 and GW26, there was increased expression of *GLI1* and *HHIP* (SHH pathway markers) in T‐tRGs and their progenies. Furthermore, genes of OBIN‐IPCs, including *GSX2, DLX2, GAD2*, and *SP9*, became evident within the Tri‐IPC cluster (Figure S10A–G, Supporting Information). Thus, YAP signaling drives the cortical E‐tRG‐derived multiciliated ependymal cell lineage,^[^ ^41^, ^42^ ^]^ whereas SHH‐SMO signaling promotes the primary‐ciliated T‐tRG‐derived Tri‐IPC cell lineage.^[^ ^13^, ^15^, ^17^, ^35^ ^]^

In summary, during the later stages of human cortical development, PyN‐IPC‐generating oRGs, ependymocyte‐generating E‐tRGs, and Tri‐IPC‐generating T‐tRGs develop distinct molecular signatures, driven by different conserved signaling pathways that mutually inhibit one another (Figure 6C). These findings reveal that ERK and PKA signaling synergistically sustain prolonged self‐renewal and neurogenic capacity in human cortical oRGs, a critical driver of cortical neurogenesis and expansion, by coordinately suppressing the gliogenic YAP/TAZ and SHH pathways.

## 3 Discussion

There are 6 main findings in this study: 1) YAP/TAZ signaling promotes mouse cortical E‐RG identity and the generation of ependymal cells while repressing both ERK/PKA and SHH signaling. 2) SHH‐SMO signaling promotes mouse cortical T‐RG identity and the generation of cortical Tri‐IPCs, and represses ERK/PKA and YAP signaling. 3) PKA signaling maintains mouse cortical N‐RG identity and suppresses both YAP and SHH signaling pathways. 4) ERK and PKA signaling mutually reinforce each other in cortical N‐RGs, collectively enhancing self‐renewal and prolonging the neurogenic period by repressing gliogenic pathways, thereby promoting increased neuronal production. 5) During cortical Tri‐IPC genesis, ERK/PKA signaling in ventral, lateral, and dorsolateral cortical RGs represses YAP signaling, thereby preventing premature ependymal differentiation and ensuring the production of Tri‐IPCs—a process primarily mediated by SHH‐SMO signaling. 6) Human cortical neurogenesis, gliogenesis, and evolutionary expansion are governed by an integrated program coordinated by the conserved ERK, PKA, YAP, and SHH pathways, as in mice. During evolution, this program was adapted in humans, whereby cortical oRGs acquired dominant ERK/PKA signaling that suppresses YAP and SHH, enhancing their self‐renewal and sustaining a prolonged neurogenic period. Our study reveals fundamental principles governing mammalian cortical development and evolution.

**Figure 7:**
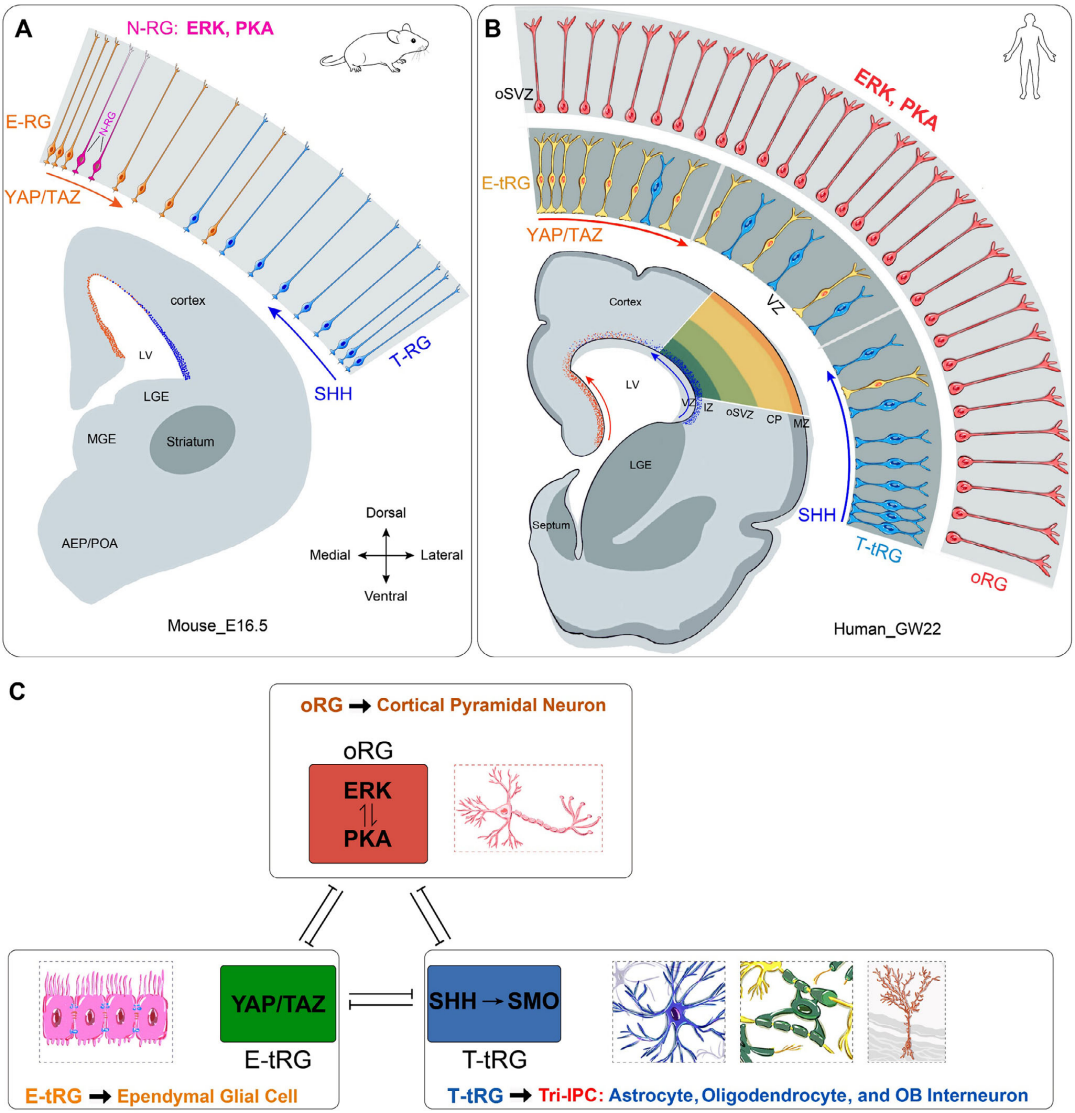
The integrated ERK, PKA, YAP/TAZ, and SHH signaling drives mammalian cortical expansion and lineage diversification. A) Cortical RG subtypes in mouse at E16.5. B) At GW22, human cortical RGs primarily consist of: oRGs in the OSVZ, generating upper‐layer PyNs; E‐tRGs in the VZ, producing cortical ependymal glial cells; T‐tRGs in the VZ, giving rise to Tri‐IPCs, which sequentially differentiate into astrocytes, oligodendrocytes, and cortically derived olfactory bulb interneurons. C) During corticogenesis, cortical RGs adopt one of three distinct developmental trajectories, each regulated by specific signaling pathways: ERK/PKA upregulation in RGs results in sustained neurogenesis with concomitant suppression of gliogenesis; YAP/TAZ upregulation in RGs results in generating ependymal cells; enhanced SHH signaling in RGs results in generating Tri‐IPCs. Note that during cortical evolution, ERK/PKA signaling becomes the dominant pathway in human cortical oRGs via a self‐reinforcing feedback loop that actively suppresses both YAP and SHH signaling. This evolutionary adaptation confers two critical advantages: an enhanced self‐renewal capacity in human cortical oRGs and a prolonged neurogenic period in RGs. More importantly, ERK signaling exhibits a rostral‐high to caudal‐low gradient in the cortex, which promotes the expansion of the prefrontal cortex. Collectively, these mechanisms drive the dramatic expansion of neuronal output, neocortical volume, and the prefrontal cortex—defining features of human cortical evolution. This also demonstrates that cortical neurogenesis, gliogenesis, and evolutionary expansion constitute an integrated biological program, rather than independent processes. This program is coordinated by ERK, PKA, YAP/TAZ, and SHH signaling, which collectively form a tripartite repressive network.

During the early regional patterning stage of corticogenesis, the development is predominantly orchestrated by WNT, FGF, and BMP signaling.^[^ ^45^, ^86^, ^87^ ^]^ This signaling network promotes RGs to generate PyNs through a precisely regulated inside‐out developmental sequence.^[^ ^1^, ^2^, ^3^, ^4^, ^5^, ^88^ ^]^ Next, the neurogenic period of cortical RGs is regulated by a combination of intrinsic molecular mechanisms and extrinsic environmental cues (**Figure** **7**). In mice, as YAP and SHH signaling pathways intensify in cortical RGs, they antagonize ERK and PKA signaling, reaching a critical threshold around E16.5. Around this stage, a subset of medial N‐RGs transitions into E‐RGs, which subsequently differentiate into ependymal cells.^[^ ^9^, ^10^, ^11^, ^12^ ^]^ Concurrently, N‐RGs in the ventral, lateral, and dorsolateral cortex transition into T‐RGs, starting the generation of Tri‐IPCs (Figure 7A).^[^ ^15^, ^16^, ^18^ ^]^

During human corticogenesis, fRGs (full‐span RGs) first undergo PyN genesis from GW8 to GW16.^[^ ^4^, ^18^, ^76^ ^]^ By GW16, following ≈9 weeks of neurogenesis, the human fetal cortex has undergone significant expansion and harbors a substantial population of PyNs that require glial cell‐derived support and nutrient supply from the developing vascular network. Concurrently, YAP and SHH signaling, including CXCL12/CXCR signaling, progressively intensify in fRG cells. To accommodate the evolutionary pressure‐driven expansion of PyN populations while concurrently meeting the heightened demand for glial cell production, human fRGs undergo a specialized division process, giving rise to oRGs and tRGs.^[^ ^4^, ^18^, ^76^ ^]^ oRGs inherit the elongated basal processes from their fRGs and exhibit elevated levels of ERK and PKA activity, which are mutually reinforcing. ERK and PKA signaling enhancement suppresses gliogenic YAP and SHH signaling, thereby maintaining the neurogenic program in oRGs (Figure 7B). In contrast, human cortical tRGs inherit the apical membrane domains from fRGs, maintaining direct contact with the lateral ventricle. Subsequently, the enhanced activation of the YAP and SHH signaling orchestrates the establishment of E‐tRGs and T‐tRGs identities, steering their differentiation into ependymal cells and Tri‐IPCs, respectively (Figure 7B,C).

The loss of FGFR or ERK function in mouse cortical RGs results in remarkably similar cortical phenotypes.^[^ ^17^, ^62^, ^63^, ^64^ ^]^ FGFR‐mediated ERK signaling plays an essential role in multiple aspects of cortical development, including neural induction, cortical regional patterning, RG cell proliferation, self‐renewal, survival, growth, and promoting *BMP7* and *HOPX* expression.^[^ ^15^, ^17^, ^43^, ^45^, ^46^, ^62^, ^63^, ^64^ ^]^ In this study, we observed that ERK and PKA signaling establish a mutually reinforcing positive feedback loop, mediated through repressing gliogenic YAP and SHH signaling, which collectively drive the expansion of the cortical RG population and extend the neurogenic period. Both the ERK and PKA signaling pathways are tightly regulated by robust negative feedback mechanisms, which effectively prevent uncontrolled signal amplification and maintain cellular signaling homeostasis.^[^ ^48^, ^49^ ^]^ Interestingly, this regulatory program of FGF‐ERK signaling exhibits remarkable evolutionary conservation, with homologous mechanisms identified in hemichordates and amphioxus, suggesting its origin dates back at least 500 million years.^[^ ^89^ ^]^ More importantly, FGF‐ERK signaling exhibits a rostral‐high to caudal‐low gradient in the cortex, which promotes the expansion of the prefrontal cortex during evolution—a hallmark feature of the human cerebral cortex. Based on these findings, we propose that ERK signaling likely plays a central role in the regulatory network driving cortical development and the expansion of PyN numbers during evolution.

This study further led to the establishment of a novel model, in which a tripartite signaling network, centered on cross‐repressive interactions among the ERK/PKA, YAP, and SHH pathways, regulates mammalian cortical neurogenesis, ependymal gliogenesis, and Tri‐IPC genesis. Furthermore, it provides foundational evidence for the basic machinery underlying cortical expansion and evolution. Our model, however, inevitably challenges the conclusions of numerous previous studies, while our data support a distinct alternative perspective. For instance, although prior work has proposed that SHH signaling drives cortical expansion and gyrification,^[^ ^90^, ^91^, ^92^, ^93^, ^94^, ^95^ ^]^ our loss‐of‐function (*Smo‐cko*) and gain‐of‐function (*dnPKA* overexpression) experiments demonstrate that SHH signaling instead plays a pivotal role in regulating the generation of cortical Tri‐IPCs. Accordingly, we conclude that SHH signaling does not directly contribute to cortical expansion. Several previous studies have concluded that YAP signaling promotes cortical expansion and gyrification.^[^ ^96^, ^97^, ^98^, ^99^, ^100^ ^]^ In contrast, our loss‐ and gain‐of‐function analyses demonstrate that YAP signaling primarily regulates cortical ependymal cell development via coordinated suppression of ERK, PKA, and SHH signaling pathways. Based on these findings, we conclude that YAP signaling does not drive cortical expansion. Numerous studies have proposed that human cortical RGs, particularly oRGs, generate cortical interneurons, OPCs, or Tri‐IPCs.^[^ ^14^, ^101^, ^102^, ^103^, ^104^, ^105^, ^106^, ^107^, ^108^, ^109^ ^]^ In contrast, our findings argue against this view, as we propose that human oRGs predominantly produce PyNs. Cortical PyN fate specification is mediated by robust PKA and ERK signaling activity in RGs, which potently suppresses gliogenic YAP and SHH signaling pathways.

In summary, this study elucidates the molecular mechanisms responsible for the specification and lineage progression of distinct cortical RG subtypes across murine and human systems. It also uncovers fundamental principles that coordinate cortical neurogenesis, gliogenesis, and expansion, which are conserved during evolution. Thereby these findings provide critical insights into the fundamental mechanisms of cortical development (Figure 7).

## 4 Experimental Section

### Animals

All procedures involving animals were approved by and performed in accordance with the guidelines of the Fudan University Shanghai Medical College Animal Ethics Committee (No. 20230301‐141). *Emx1‐Cre* (JAX no. 0 05628),^[^ ^65^ ^]^ *hGFAP‐Cre* (JAX no. 0 04600),^[^ ^66^ ^]^ *Rosa^Mek1DD^* (JAX no. 01 2352),^[^ ^110^ ^]^ *Smo flox* (JAX no.004526),^[^ ^111^ ^]^ *Map2k1* (exon 2) floxed, and *Map2k2* (exon 4–9) floxed mice were described previously.^[^ ^17^ ^]^ *SuperHippo* mice were provided by Professor Faxing Yu at Fudan University.^[^ ^61^ ^]^ The day of detecting a vaginal plug was designated as E0.5. The day of birth was designated as P0. The sexes of the embryonic and early postnatal mice were not determined. For immunostaining analysis, normally, brains from 4 to 5 independent experiments were processed (*n* values refer to numbers of brains analyzed).

### Tissue Preparation

Embryos were isolated from deeply anesthetized pregnant mice. Brains were dissected and fixed overnight in 4% paraformaldehyde (PFA) pretreated with diethylpyrocarbonate (DEPC). Postnatal mice were deeply anesthetized and transcardially perfused with phosphate‐buffered saline (PBS), followed by 4% PFA. All brains were postfixed overnight in 4% PFA at 4 °C and subsequently dehydrated in 30% sucrose for at least 24 h. The brains were then embedded in O.C.T. compound (Sakura Finetek) and stored at ‐80 °C. For analysis, mouse brains were sectioned into 20‐µm‐thick slices.

### Plasmid Construction

*pCAG‐ires‐GFP* plasmid was from Addgene (Addgene #11 150). The mouse *dnPKA* cDNA represents a dominant‐negative form of PKA.^[^ ^112^ ^]^ This variant is derived from PKA‐RIa, which is encoded by the *Prkar1a* gene, and is characterized by mutations at the cAMP binding sites, rendering it unresponsive to cAMP. The *dnPKA* cDNA was subsequently cloned into the *pCAG‐GFP* vector to generate *pCAG‐dnPKA‐ires‐GFP* plasmids.

### In Utero Electroporation (IUE)

In utero electroporation was performed as described previously.^[^ ^13^ ^]^ Briefly, pregnant mice were anesthetized using an animal anesthesia machine with isoflurane. A plasmid solution (final concentration: 1–2 µg µL^−1^ for each plasmid, 0.5 µL per embryo), mixed with 0.05% Fast Green (Sigma), was injected into the lateral ventricle of embryos using a beveled glass micropipette. Five electrical pulses (duration: 50 ms) were delivered at optimized voltages across the uterine wall, with a 950 ms interval between pulses, using a pair of 7‐mm platinum electrodes (BTX, Tweezertrode 45‐0488, Harvard Apparatus) connected to an electroporator (BTX, ECM830). The applied voltages were adjusted according to embryonic age: 33 V for E14, 35 V for E15, and 38 V for E18. Electroporated brains were analyzed at specified time points, as indicated in the main text.

### FlashTag Labeling

FlashTag labeling was performed via in utero injection, and the FlashTag‐labeled live cells were subsequently isolated using fluorescence‐activated cell sorting (FACS).^[^ ^113^, ^114^ ^]^ To label embryonic day 15.5 (E15.5) cortical progenitors, FlashTag‐CellTrace Yellow solution (Life Technologies, #C34567; 0.5 µL of 10 mm) was injected into the lateral ventricles of *SuperHippo* with or without *hGFAP‐Cre* and *Emx1‐Cre*, mice at E15.50, with analysis performed at E16.5. To label E17.0 progenitors, CellTrace Yellow solution was injected into the mouse lateral ventricles of *Smo^F/F^* and *hGFAP‐Cre; Smo^F/F^* mice at embryonic day 17.0 (E17.0), with analysis performed at E18. To label P0 progenitors, the CellTrace solution was injected into the lateral ventricles of the following groups at postnatal day 0 (P0), with analysis conducted at P2: Control (*SuperHippo* without *Cre*), *hGFAP‐Cre; SuperHippo*, *Smo^F/F^*, *hGFAP‐Cre; Smo^F/F^*, *hGFAP‐Cre; Map2k1/2‐dcko*, and *Emx1‐Cre; Map2k1/2‐dcko* mice. Mice were sacrificed, and brains were immediately removed and submerged in fresh ice‐cold Hanks’ balanced salt solution (HBSS; Gibco, 14175‐095). Cortices were dissected, dissociated into single‐cell suspensions, and subjected to FACS to purify FlashTag‐labeled cells. Single‐cell RNA sequencing (scRNA‐Seq) was performed on FACS‐sorted cells using the 10X Genomics platform.

### Immunohistochemistry

Immunohistochemistry was performed on 20 µm coronal sections of mouse brains using standard protocols. Sections were rinsed with Tris‐buffered saline (TBS; 0.01 m Tris‐HCl, 0.9% NaCl, pH 7.4) for 10 min, permeabilized with 0.5% Triton‐X‐100 in TBS for 30 min at room temperature (RT), and blocked in a solution of 5% donkey serum and 0.5% Triton‐X‐100 in TBS (pH 7.2) for 2 h. After blocking, sections were incubated with primary antibodies diluted in blocking buffer overnight at 4 °C. The following day, sections were washed three times with TBS (10 min each) and incubated with secondary antibodies (1:500; Jackson ImmunoResearch) for 2 h in the dark at RT. After three additional TBS washes (10 min each), sections were counterstained with 4′,6‐diamidino‐2‐phenylindole (DAPI; Sigma, 200 ng mL^−1^) for 2 min. All primary antibodies utilized in this investigation, including their sources, catalog numbers, host species, and working dilutions, are detailed in Table S1 (Supporting Information).

### scRNA‐Seq

Briefly, mouse cortices were dissociated into single‐cell suspensions, and selected samples were screened by FACS prior to single‐cell sequencing. scRNA‐Seq libraries were generated using the Chromium droplet‐based platform (10X Genomics) according to the manufacturer's instructions (manual document part number: CG00052 Rev C). Purified cDNA libraries were quantified using an Agilent 2100 Bioanalyzer and sequenced on an Illumina NovaSeq 6000 platform. This study generated and systematically characterized a collection of 11 novel scRNA‐Seq datasets, the complete details of which are presented in Table S2 (Supporting Information).

scRNA‐Seq data derived from human cortical tissues at GW22, GW23, and GW26 were obtained from a previously published study.^[^ ^82^ ^]^ For detailed methodological procedures, readers were to the original publication.^[^ ^82^ ^]^

### scRNA‐Seq Data Analysis

scRNA‐Seq reads were aligned to the mm10 reference genome and quantified using “cellranger count” (10x Genomics, v.7.1.0). Count data were further processed using the “Seurat” R package (v.5.1.0). Genes detected in fewer than three cells, cells with fewer than 500 detected genes, and cells with more than 10% mitochondrial gene content were filtered out. The selection of highly variable genes was performed using the Variance Stabilizing Transformation (VST) method, which models mean‐dependent technical noise and is robust to the influence of low‐complexity cells. After filtering, the number of cells in each dataset was as follows:

E15.0 cortex: *Map2k1/2* (without *Cre*) 12128 cells, 2673 genes per cell, *Emx1‐Cre; Map2k1/2‐dcko* mice, 9291 cells, 2850 genes per cell; E16.5 FACS cortical progenitors: wild‐type 14 800 cells, 1504 genes per cell, *hGFAP‐Cre; SuperHippo* 13171 cells, 1544 genes per cell; E16.5 FACS cortical progenitors: *SuperHippo* without *Emx1‐Cre* 13194 cells, 2582 genes per cell, *Emx1‐Cre; SuperHippo* mice, 14 641 cells, 2616 genes per cell; E18.0 FACS cortical progenitors labeled by dnPKA‐GFP in *hGFAP‐Cre; Smo^F/F^* 11508 cells, 2369 genes per cell; P2 FACS cortical progenitors: control (*Smo^F/F^*), 14292 cells, 2498 genes per cell; *hGFAP‐Cre; Smo^F/F^* mice, 7694 cells, 2681 genes per cell; control (*SuperHippo* heterozygotes without *hGFAP‐Cre*), 12 054 cells, 2258 genes per cell; *hGFAP‐Cre; SuperHippo* mice, 11 539 cells, 2120 genes per cell. These 11 novel scRNA‐Seq data have been deposited in the GEO under the accession number GSE293205.

Gene expression data were normalized using the global‐scaling method “LogNormalize.” The top 2000 highly variable genes were identified with the FindVariableFeatures function using the vst method. To minimize the influence of the cell cycle on clustering and dimensionality reduction, cell cycle‐associated gene sets were used to score the cell phase of each cell with the CellCycleScoring function, and the difference between G2M and S phase scores was regressed out using the ScaleData function. The scaled z‐scored residuals were used for Principal Component Analysis (PCA). Statistically significant principal components, identified through a resampling test, were retained for FindNeighbors and FindClusters analysis. Uniform Manifold Approximation and Projection (UMAP) was used for visualization of cell clustering. Differentially expressed genes (DEGs) among clusters were identified using the Wilcoxon rank sum test, comparing cells in each cluster against all other cells. Genes were considered statistically significant only if they achieved a false discovery rate (FDR) adjusted *P*‐value of < 0.05. Additionally, most genes presented in the present study were consistently identified by both knockout methods—specifically, *Emx1‐Cre* and *hGFAP‐Cre* mediated knockout conditions at equivalent embryonic time points. A key advantage of this strategy is its ability to pinpoint the true downstream targets of a gene or signaling pathway following genetic perturbation.

### Image Acquisition and Analysis

All brain section images in this study were acquired using an Olympus VS120 Automated Slide Scanner (10X, 20X) and an FV3000 confocal microscope system (40X). Images were processed using Adobe Photoshop for clarity, false colorization, and overlay as needed. Both Adobe Photoshop and Adobe Illustrator were used to adjust images without altering the original data.

### Quantification and Statistical Analysis

Quantitative images were acquired using the Olympus VS120 Automated Slide Scanner (10X or 20X). Statistical analyses were performed using GraphPad Prism 10 and IBM SPSS Statistics 29.0. For each experiment, at least four control or mutant mouse samples were analyzed. The following quantifications were performed:

1. Quantification of GSX2‐, OLIG2‐, and EGFR‐positive cells in the cortical VZ/SVZ (width: 1200 pixels; height: 800 pixels) at E18 in *Smo^F/F^*, *Smo^F/F^+ dnPKA*, *hGFAP‐Cre; Smo^F/F^*, and *hGFAP‐Cre; Smo^F/F^+ dnPKA* mouse brains (see Figure 3D,E).
2. Quantification of FOXJ1‐ and CRYAB‐positive cells in the cortex of control and *Emx1‐Cre; Map2k1/2‐dcko* mice at P0. FOXJ1‐ and CRYAB‐positive cells in the dorsal cortical VZ/SVZ were counted. Four brain slices from corresponding locations were analyzed per sample (see Figure 4D,E).
3. Quantification of GSX2^+^ and OLIG2^+^ cells in the cortex of control and *hGFAP‐Cre; SuperHippo* mice. Four brain slices from corresponding positions were selected per sample, and GSX2^+^ and OLIG2^+^ cells in the entire VZ/SVZ region were counted (see Figure 2D,E; and Figure S2D,E, Supporting Information).
4. Quantification of cortical area at P21 in control and *Emx1‐Cre; Map2k1/2‐dcko* mice. Six brain slices from corresponding positions were analyzed per sample, and the cortical area was measured using Photoshop (see Figure S6A–C, Supporting Information).
5. Quantification of EOMES^+^ cells in the cortical VZ (width: 300 pixels; height: 300 pixels) of control and *Emx1‐Cre; Rosa^Mek1DD^* mice at E13.5. Four brain slices from corresponding positions were analyzed per sample (see Figure S6D,E, Supporting Information).
6. Quantification of cortical area at P76 in control and *Emx1‐Cre; Rosa^Mek1DD^* mice. Six brain slices from corresponding positions were analyzed per sample, and the cortical area was measured using Photoshop (see Figure S6F–H, Supporting Information).
7. Quantification of FOXJ1‐ and CRYAB‐positive cells in the cortex of control and *Emx1‐Cre; Map2k1/2‐dcko* mice at P2. FOXJ1‐ and CRYAB‐positive cells in the dorsal cortical VZ/SVZ were counted. Four brain slices from corresponding locations were analyzed per sample (see Figure 5C,D). Data are presented as mean ± SEM. Statistical significance for single comparisons was determined using unpaired *t*‐tests. A *P*‐value < 0.05 was considered statistically significant (*P < 0.05, **P < 0.01, ***P < 0.001).

## Conflict of Interest

The authors declare no conflict of interest.

## Author Contributions

Z.Z., Z.X., T.F., and J.L. contributed equally to this work. Conceptualization: Z.Y. Data curation: T.F., Z.Z., Z.X., and J.L. Funding acquisition: Z.Z., Z.X., T.M., and Z.Y. Investigation: Z.Z., Z.X., T.F., J.L., F.Y., C.Y., W.Z., Z.S., Y.G., M.S., Z.L., J.D., and X.L. Resources: Z.Y. Supervision: Z.Y. Writing: Z.Y. All of the authors contributed to reviewing and editing of the manuscript.

## Supporting information



Supporting Information

## Data Availability

The datasets (scRNA‐Seq from 11 samples, Table S2, Supporting Information) generated during the current study are available in the Gene Expression Omnibus (GEO: GSE293205). The E18.0 *Smo^F/F^* (control) and *hGFAP‐Cre; Smo^F/F^* mouse cortex scRNA‐Seq data were used from our previous study (GSE221389).^[^ ^35^ ^]^ The P2 *Emx1‐Cre; Map2k1/2‐dcko* and P2 *hGFAP‐Cre; Map2k1/2‐dcko* mouse cortex scRNA‐Seq data were used from the previous studies (GSE274547). Bulk RNA‐Seq data from the SVZ of wild‐type control mice (*n* = 3) and tumor cells derived from ≈P20 mice transfected with YAP‐M at E14.5 (*n* = 4), and ChIP‐Seq and Cut&Run data for YAP1 and H3K27ac in both YAP‐M‐induced tumor cells and YAP1‐expressing mouse neural stem cells were used in previous studies (GSE181867).^[^ ^67^ ^]^ scRNA‐Seq data derived from human cortical tissues at GW22, GW23, and GW26 were obtained from a previously published study (GSE162170).^[^ ^82^ ^]^
