# Supplementary material for: Integrated ERK‐PKA‐YAP/TAZ‐SHH Signaling Orchestrates Cortical Radial Glia Identity and Lineage Diversification
Source: Adv Sci (Weinh). 2025 Nov 12;13(5):e13571. doi: 10.1002/advs.202513571 (PMC12850263; doi:10.1002/advs.202513571)
Supplement: Supplementary file 1 — Supporting Information [file ADVS-13-e13571-s001.pdf]

Supporting Information

Integrated ERK-PKA-YAP/TAZ-SHH Signaling Orchestrates Cortical Radial Glia Identity and Lineage Diversification

Zhuangzhi Zhang[1]†, Zhejun Xu[1]†, Tongye Fu[1]†, Jialin Li[1]†, Feihong Yang[1], Chuannan Yang[1], Wenhui Zheng[1], Zizhuo Sha[1], Yanjing Gao[1], Mengge Sun[1], Zhenmeiyu Li[1], Jing Ding[1], Xiaosu Li[1], Zhengang Yang*[1]

†These authors contributed equally to this work

[1]State Key Laboratory of Brain Function and Disorders, Ministry of Education Frontiers Center for Brain Science, Institutes of Brain Science, and Department of Neurology, Zhongshan Hospital, Fudan University, Shanghai 200032, China

*Zhengang Yang: yangz@fudan.edu.cn

This file includes:

Figure S1-S10
Table S1-S2

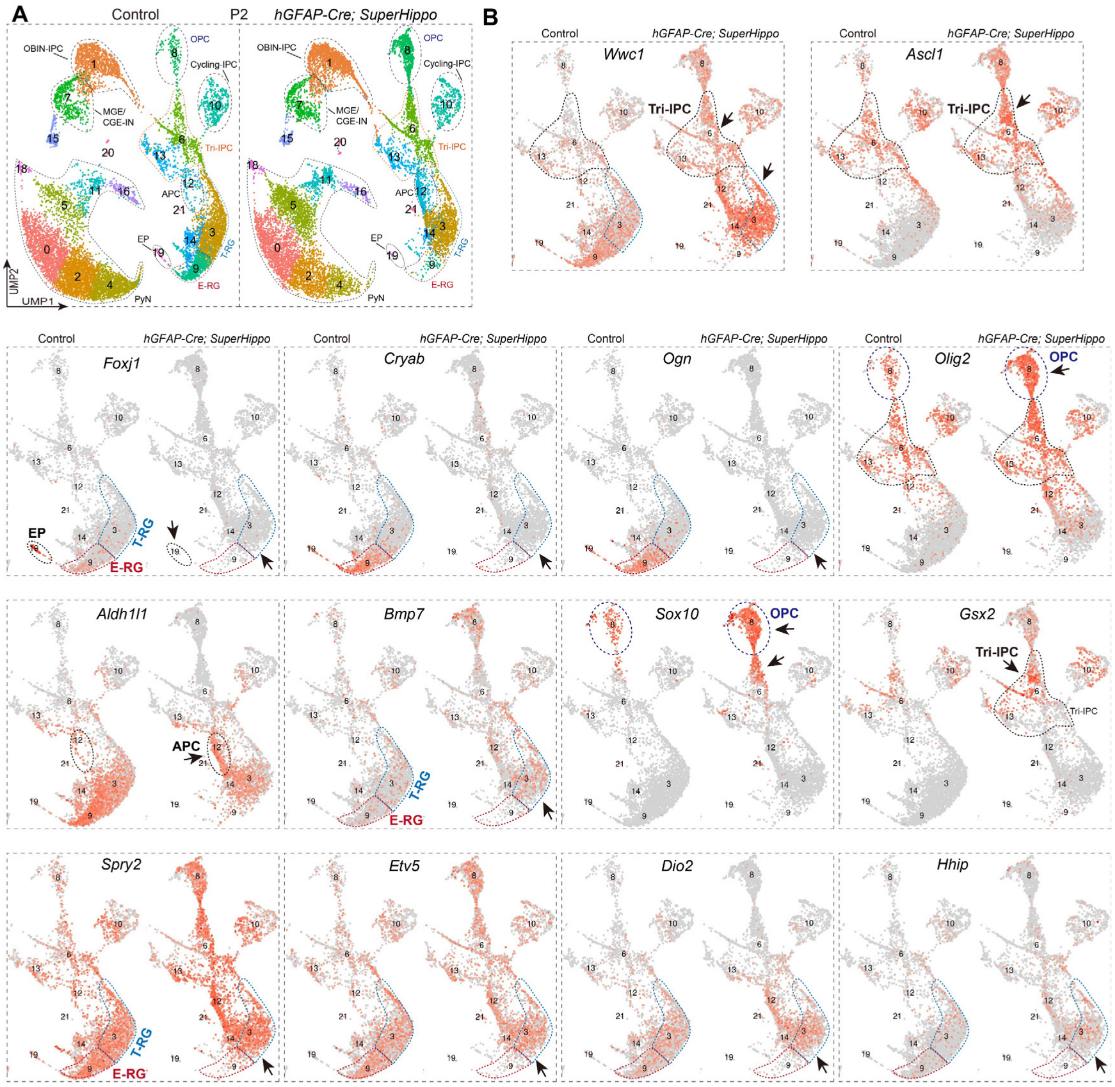

Figure S1. Loss of YAP signaling results in the depletion of mouse cortical E-RGs and ependymal cells. A) UMAP of scRNA cells colored by cluster (from **Figure 3A**). **B)** UMAP plots showing *Wwc1* gene expression was increased in *hGFAP-Cre; SuperHippo* (*Wwc1*-derived *SuperHippo* minigene) mice at P2 (arrows). Note that loss of YAP signaling results in the depletion of cortical E-RGs and the loss of early ependymal marker gene expression in cortical T-RGs, including *Foxj1, Cryab*, and *Ogn* (arrows). In contrast, there is the upregulation of genes related to Tri-IPCs (*Olig2*), APCs (*Aldh1l1*), OPCs (*Sox10*), OBIN-IPCs (*Gsx2*), ERK signaling (*Bmp7*, *Spry2*, and *Etv5*), PKA signaling (*Dio2*), and SHH signaling (*Hhip*). Tri-IPCs, tripotential intermediate progenitor cells; PyN, cortical glutamatergic pyramidal neuron; PyN-IPC, PyN intermediate progenitor cells; APC, astrocyte-IPCs; OPC, oligodendrocyte-IPCs; OBIN-IPC, IPCs for cortically derived olfactory bulb interneuron. E-RG, ependymocyte-generating RG; T-RG, Tri-IPC-generating RG; EP, ependymal cell; CGE-IN, caudal ganglionic eminence interneuron; MGE-IN, medial ganglionic eminence interneuron.

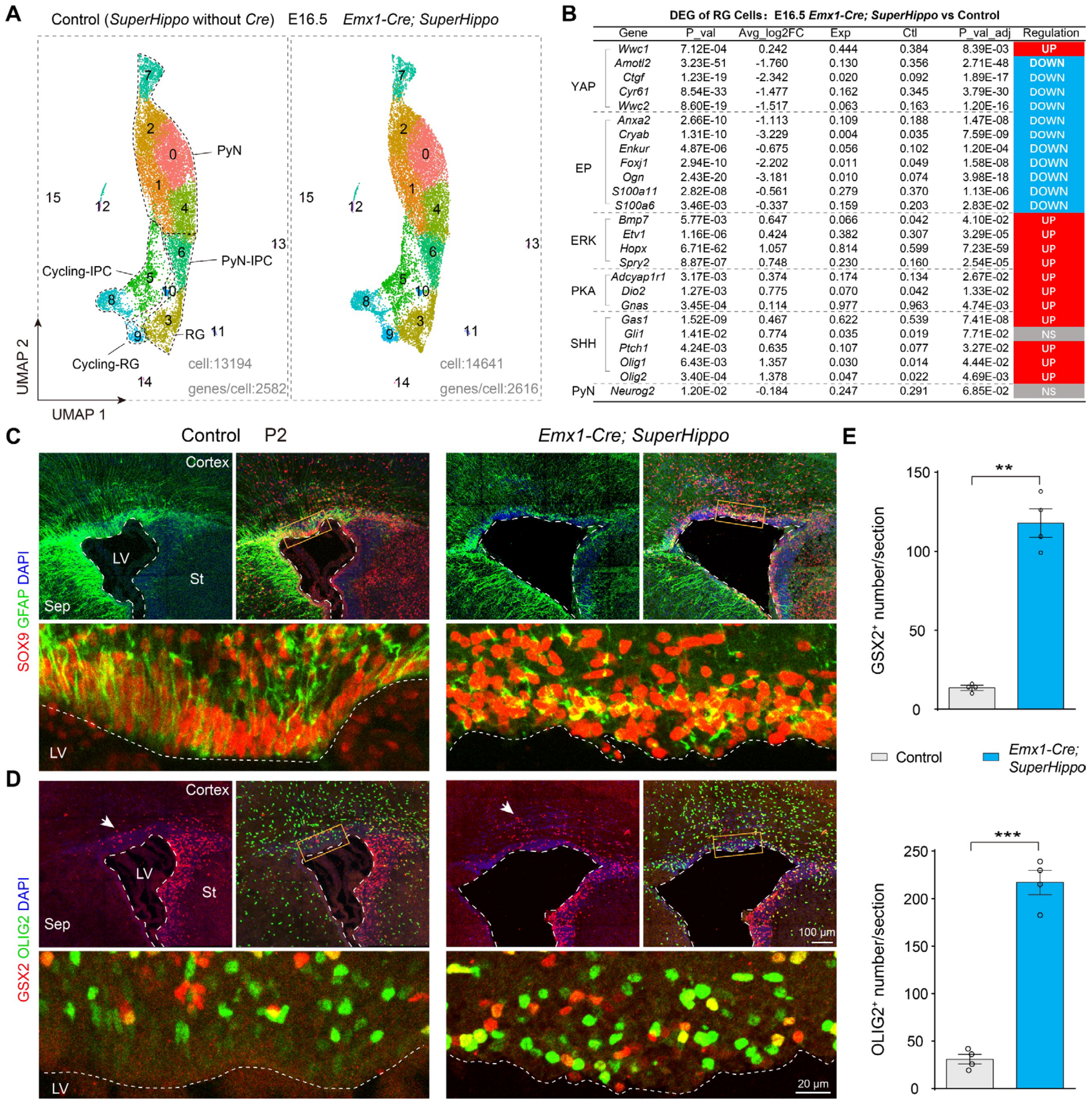

	Gene	P_val	Avg_log2FC	Exp	Ctl	P_val_adj	Regulation
YAP	*Wwc1*	7.12E-04	0.242	0.444	0.384	8.39E-03	UP
	Amotl2	3.23E-51	-1.760	0.130	0.356	2.71E-48	DOWN
	Ctgf	1.23E-19	-2.342	0.020	0.092	1.89E-17	DOWN
	Cyr61	8.54E-33	-1.477	0.162	0.345	3.79E-30	DOWN
	Wwc2	8.60E-19	-1.517	0.063	0.163	1.20E-16	DOWN
EP	*Anxa2*	2.66E-10	-1.113	0.109	0.188	1.47E-08	DOWN
	Cryab	1.31E-10	-3.229	0.004	0.035	7.59E-09	DOWN
	Enkur	4.87E-06	-0.675	0.056	0.102	1.20E-04	DOWN
	Foxj1	2.94E-10	-2.202	0.011	0.049	1.58E-08	DOWN
	Ogn	2.43E-20	-3.181	0.010	0.074	3.98E-18	DOWN
	S100a11	2.82E-08	-0.561	0.279	0.370	1.13E-06	DOWN
	S100a6	3.46E-03	-0.337	0.159	0.203	2.83E-02	DOWN
ERK	*Bmp7*	5.77E-03	0.647	0.066	0.042	4.10E-02	UP
	Etv1	1.16E-06	0.424	0.382	0.307	3.29E-05	UP
	Hopx	6.71E-62	1.057	0.814	0.599	7.23E-59	UP
	Spry2	8.87E-07	0.748	0.230	0.160	2.54E-05	UP
PKA	*Adcyap1r1*	3.17E-03	0.374	0.174	0.134	2.67E-02	UP
	Dio2	1.27E-03	0.775	0.070	0.042	1.33E-02	UP
	Gnas	3.45E-04	0.114	0.977	0.963	4.74E-03	UP
SHH	*Gas1*	1.52E-09	0.467	0.622	0.539	7.41E-08	UP
	Gli1	1.41E-02	0.774	0.035	0.019	7.71E-02	NS
	Ptch1	4.24E-03	0.635	0.107	0.077	3.27E-02	UP
	Olig1	6.43E-03	1.357	0.030	0.014	4.44E-02	UP
	Olig2	3.40E-04	1.378	0.047	0.022	4.69E-03	UP
PyN	*Neurog2*	1.20E-02	-0.184	0.247	0.291	6.85E-02	NS

Figure S2. YAP signaling represses ERK, PKA, and SHH pathway activities in cortical RGs. A) UMAP of scRNA cells colored by cluster. **B)** scRNA-Seq analysis revealed differentially expressed genes (DEG) in E16.5 mouse cortical RGs (cluster 3 in **A**) of *Emx1-Cre; SuperHippo* mice relatively to controls (*SuperHippo* mice without *Emx1-Cre*). The more pronounced phenotypes in *Emx1-Cre; SuperHippo* mice compared to *hGFAP-Cre; SuperHippo* mice at E16.5 (see Figure 1F) can be attributed to their earlier onset of Cre expression. Specifically, *Emx1-Cre* is active in cortical neuroepithelial cells starting at E10.5, whereas *hGFAP-Cre* begins driving expression in cortical RGs only around E13.0. **C-E)** At P2, immunostaining reveals disruption of the cortical ventricular surface and the loss of apical SOX9- and GFAP-expressing immature ependymal cells in the *Emx1-Cre; SuperHippo* cortex. Consistent with the elevated SHH signaling activity in cortical RGs, quantitative analysis demonstrated a significant expansion of GSX2- and OLIG2-expressing progenitors in the cortical VZ/SVZ of E*mx1-Cre; SuperHippo* mice.

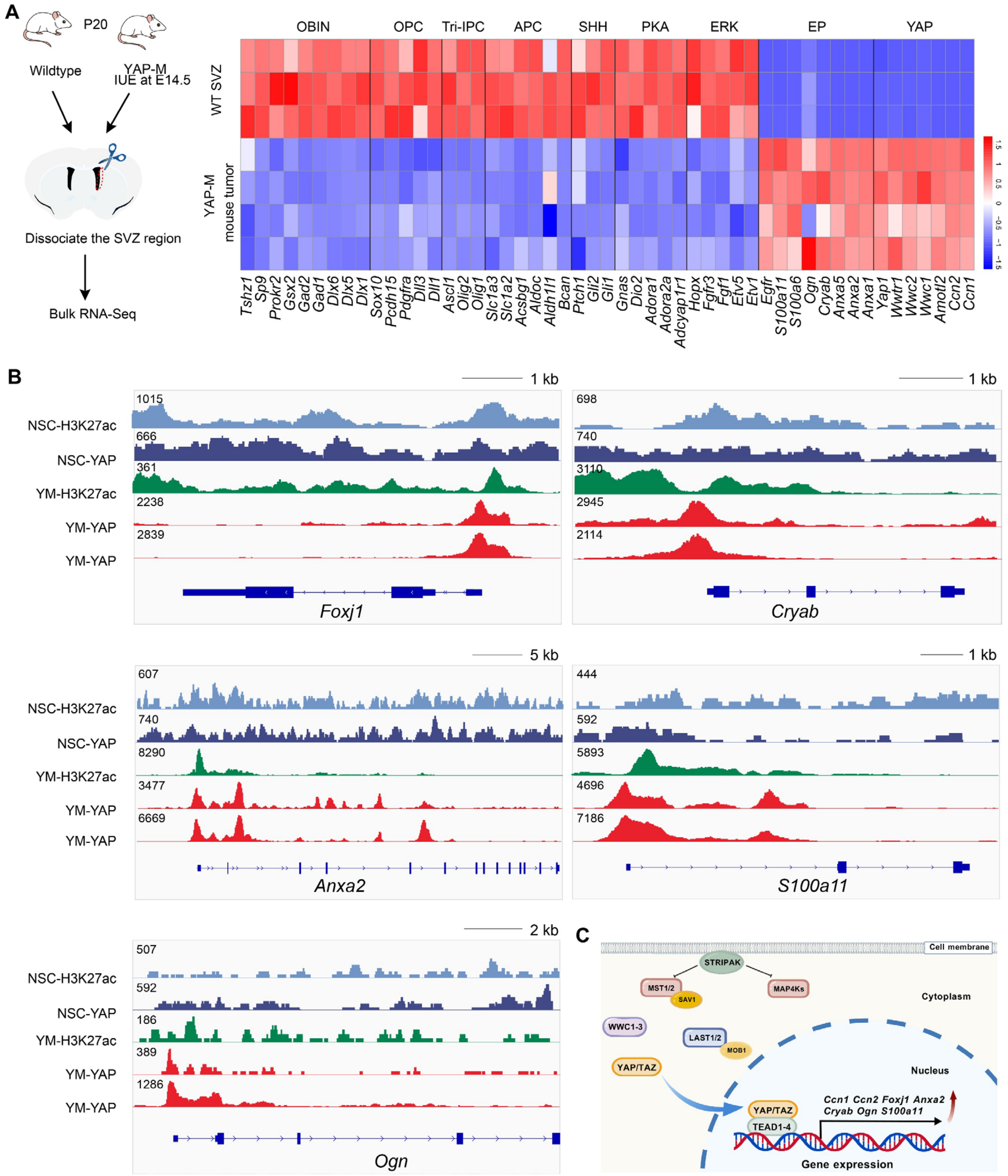

Figure S3. YAP signaling promotes the cortical E-RG cell lineage by directly activating key transcriptional regulators of ependymal development. A) Bulk RNA-Seq reanalysis. Heatmap of differentially expressed genes between the SVZ cells of wild-type control mice (n=3) and the tumor cells derived from ~P20 mice transfected with YAP-M at E14.5 (n=4 independent replicates). Note that the YAP-M fusion oncogene markedly upregulated YAP signaling components and early ependymal markers, while strongly suppressing key pathway genes (ERK, PKA, SHH) and cortical progenitor markers (Tri-IPCs, APCs, OPCs, OBIN-IPCs). **B, C)** ChIP-Seq analysis showing YAP1 binding and H3K27ac modification profiles on early ependymal cell marker gene loci in YAP-M tissue and YAP-expressing wild type neural stem cells from P20 mice.

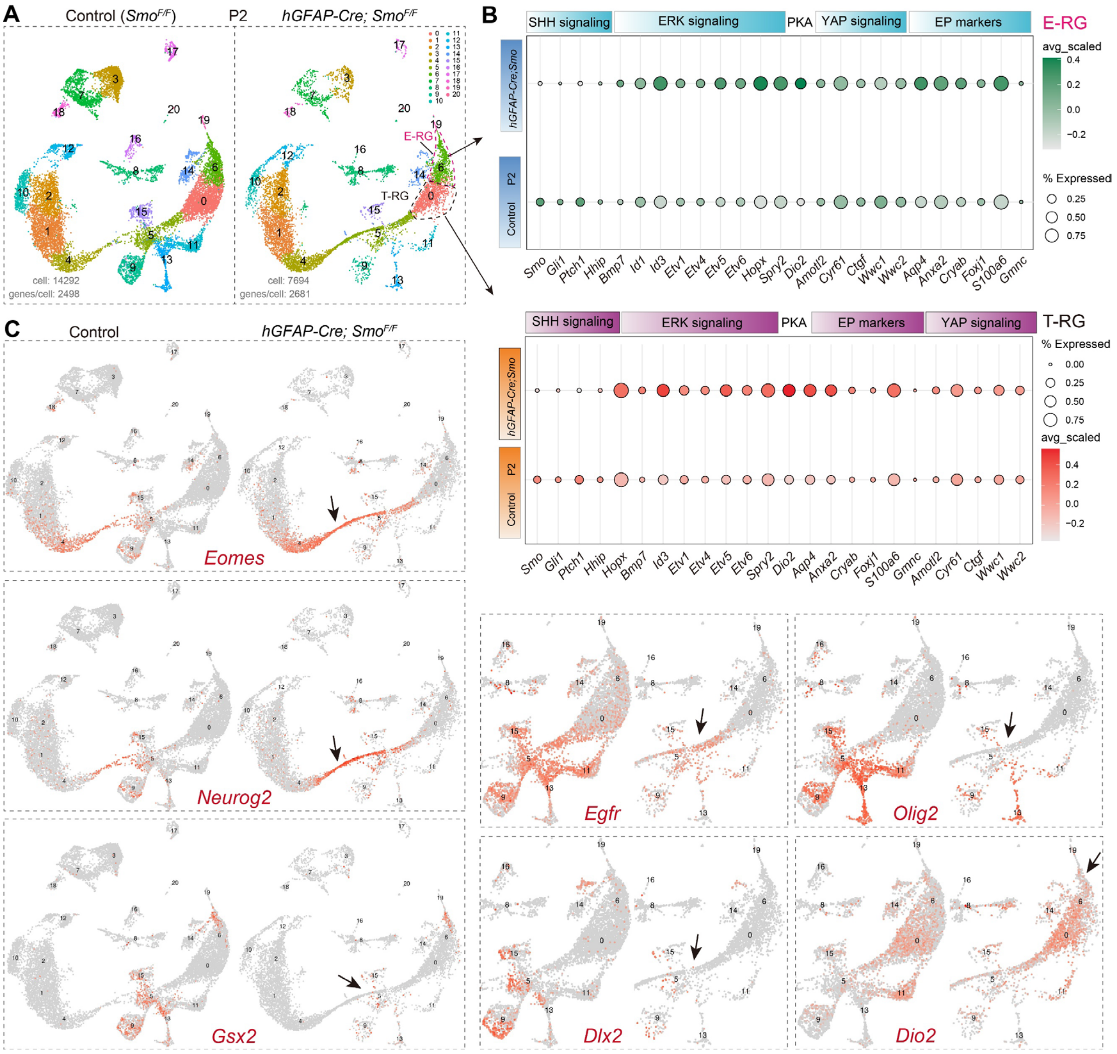

Figure S4. SHH-SMO signaling inhibits PKA, ERK, and YAP signaling pathways. **A)** UMAP of scRNA cells colored by cluster. **B)** Bubble plot showing differentially expressed genes (DEG) in cortical E-RGs and T-RGs in control (*Smo*$^{F/F}$) and *hGFAP-Cre; Smo*$^{F/F}$ mice at P2. **C)** UMAP plots showing differentially expressed genes in the cortex of control (*Smo*$^{F/F}$) and *hGFAP-Cre; Smo*$^{F/F}$ mice at P2. In the absence of *Smo* function, *Eomes, Neurog2* and *Dio2* expression were increased in cortical RGs and/or progenitors, whereas *Gsx2*, *Egfr*, *Olig2*, and *Dlx2* expression (arrows) were significantly reduced.

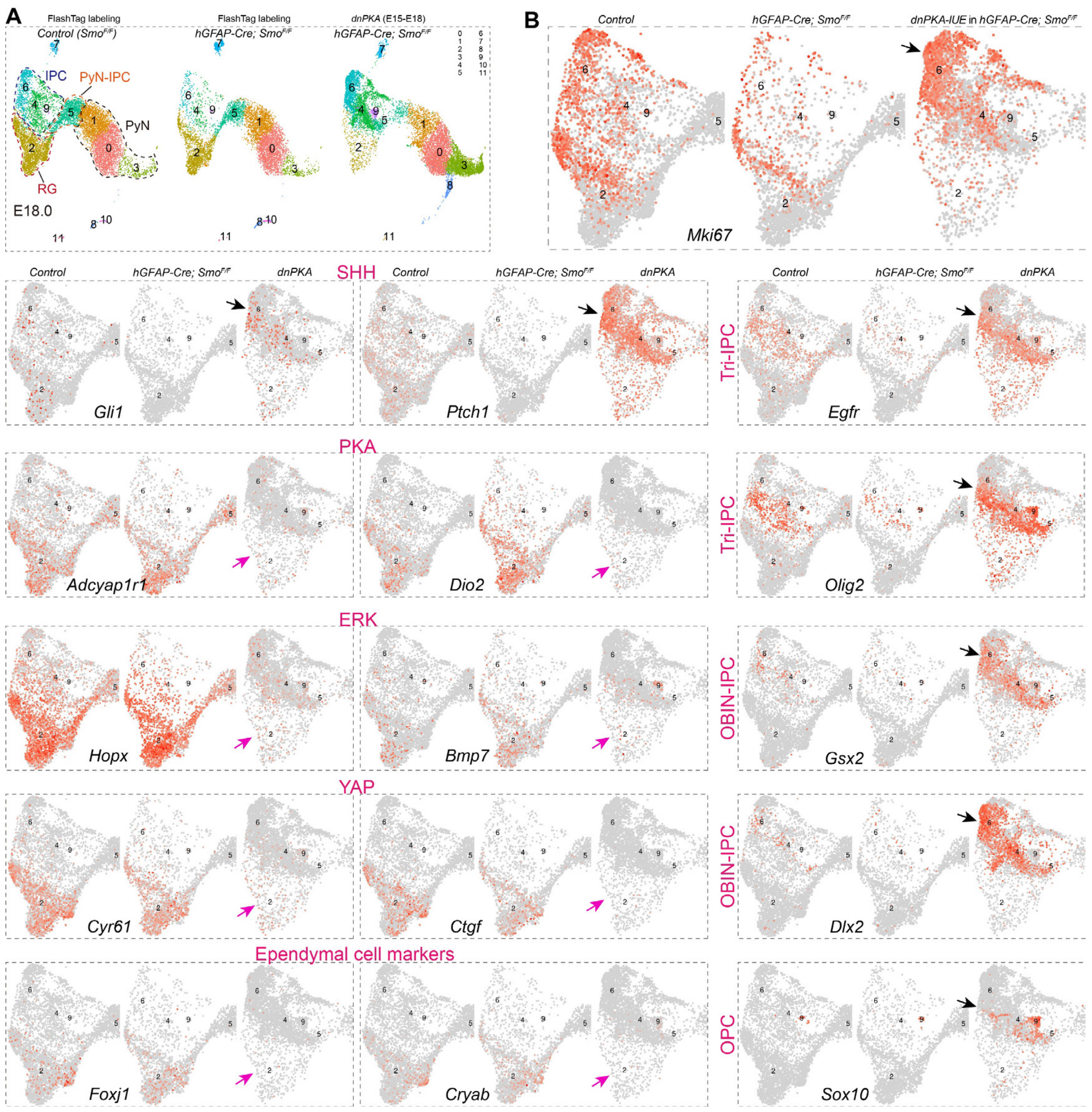

Figure S5. PKA signaling strongly inhibits SHH-SMO signaling. A) UMAP of scRNA cells colored by cluster (from **Figure 4B**). **B)** UMAP plots showing individual gene expression in the cortex of *Smo*$^{F/F}$*, hGFAP-Cre; Smo*$^{F/F}$*,* and *hGFAP-Cre; Smo*$^{F/F}$ mouse at E18.0 with IUE of *dnPKA* at E15.0. The complete loss of PKA in cortical RGs resulted in a significant increase in SHH signaling (*Gli1* and *Ptch1*), even in the absence of *Smo* function. This is accompanied by the depletion of PKA (*Adcyal1r1* and *Dio2*), ERK (*Hopx* and *Bmp7*), and YAP (*Cyr61* and *Ctgf*) signaling, as well as the loss of early ependymal marker gene (*Foxj1* and *Cryab*) expression. In contrast, there is a significant upregulation of genes associated with Tri-IPCs (*Egfr* and *Olig2*), OBIN-IPCs (*Gsx2* and *Dlx2*), and OPCs (*Sox10*) (arrows).

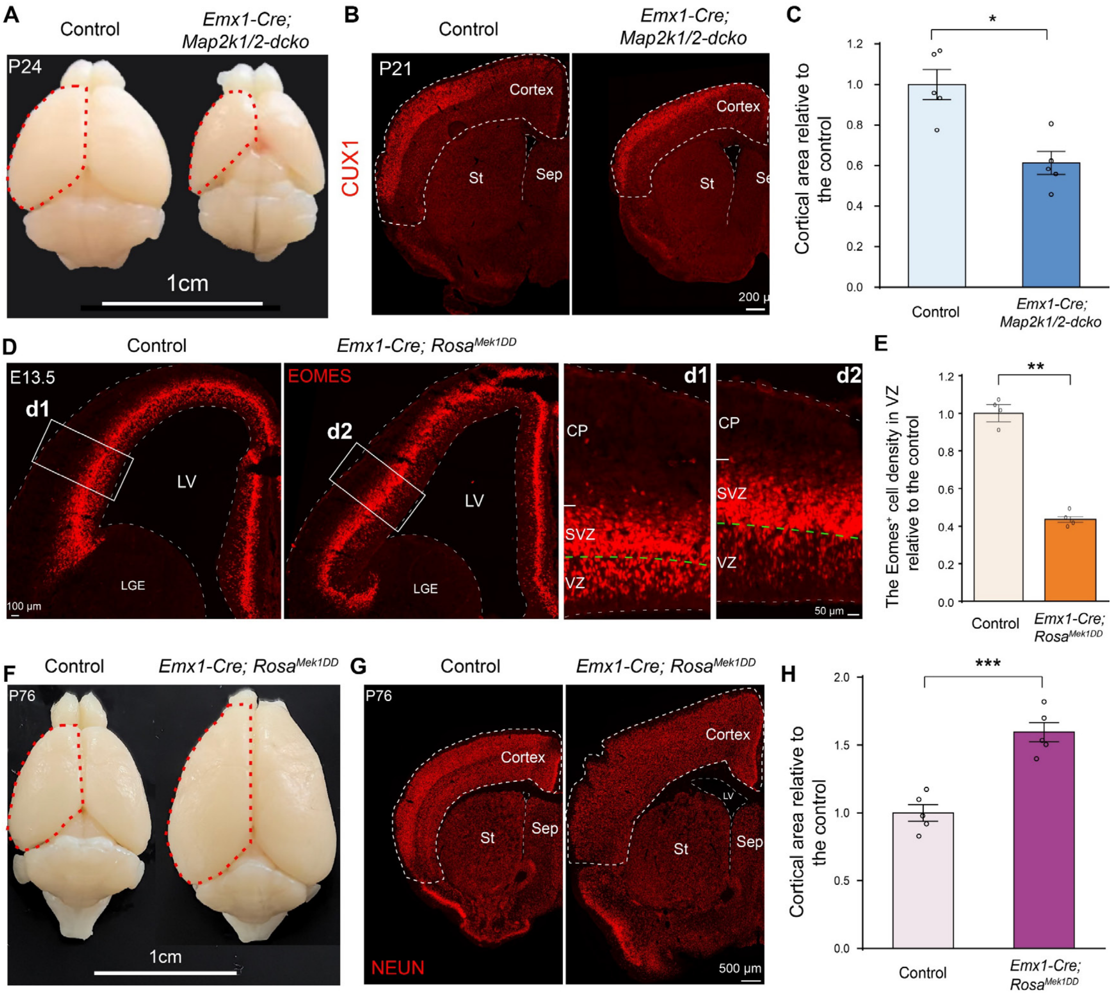

Figure S6. Loss and gain of ERK function from E10.5 cortical RGs result in pronounced microcephaly and macrocephaly, respectively. A) Whole mount images of control and *Emx1-Cre; Map2k1/2-dcko* brains. **B)** Immunostaining for CUX1 revealed fewer upper-layer PyNs and a smaller cortical area in *Emx1-Cre; Map2k1/2-dcko* cortices. **C)** Quantification of the cortical area within the cortical plate in control and *Emx1-Cre; Map2k1/2-dcko* mice. **D, E)** Immunostaining for EOMES, a marker of PyN-IPCs, revealed fewer EOMES-expressing cells in the VZ of *Emx1-Cre; RosaMEK1DD* mice at E13.5, suggesting an expansion of cortical RGs and restricted neuronal differentiation following enhanced ERK signaling. LGE, Lateral ganglionic eminence. **F)** Whole mount images of control and *Emx1-Cre; RosaMEK1DD* brains at P76. **G)** Immunostaining for NEUN, a marker of neurons. **H)** Quantification revealed a larger cortical area in *Emx1-Cre; RosaMEK1DD* mice at P76.

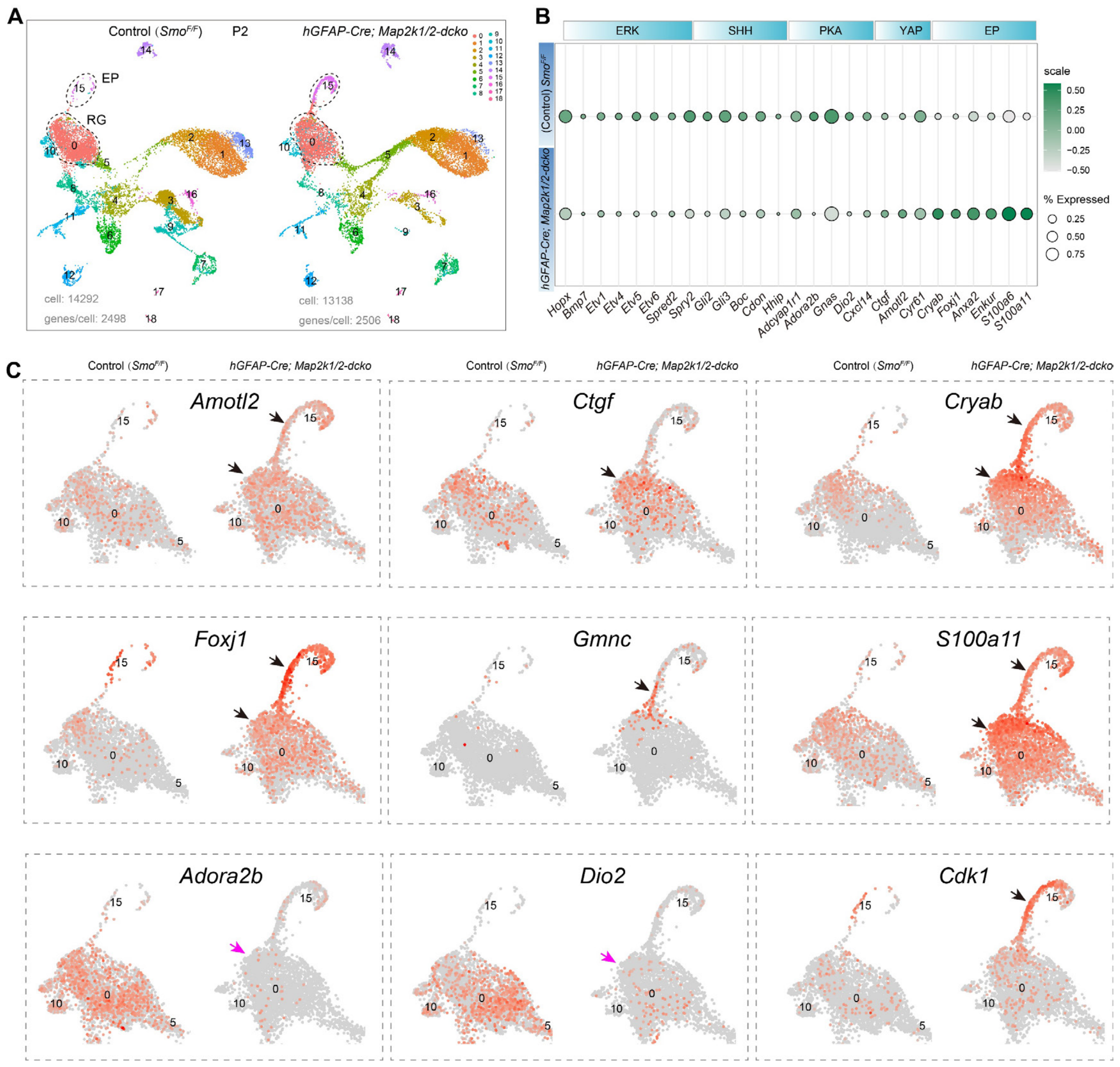

Figure S7. ERK signaling sustains PKA signaling and inhibits YAP signaling in cortical RGs. A) UMAP showing annotated cell clusters based on scRNA-Seq analysis of P2 control and *hGFAP-Ce; Map2k1/2-dcko* cortical cells. **B)** Bubble plot showing differentially expressed genes in cortical RGs (cluster 0 in **A**) of *hGFAP-Cre; Map2k1/2-dcko* mice compared to controls at P2. **C)** UMAP plots showing differentially expressed genes of YAP and PKA signaling pathway and early ependymal cell marker genes. At P2, the cortical RGs and ependymal cells of *hGFAP-Cre; Map2k1/2-dcko* mice exhibited a pronounced shift in signaling pathways compared to controls. This was characterized by a significant upregulation of YAP target genes (*Amotl2, Ctgf, Cryab, Foxj1, Gmnc, S100a11*) coupled with a marked downregulation of genes associated with PKA signaling (*Adora2b* and *Dio2*).

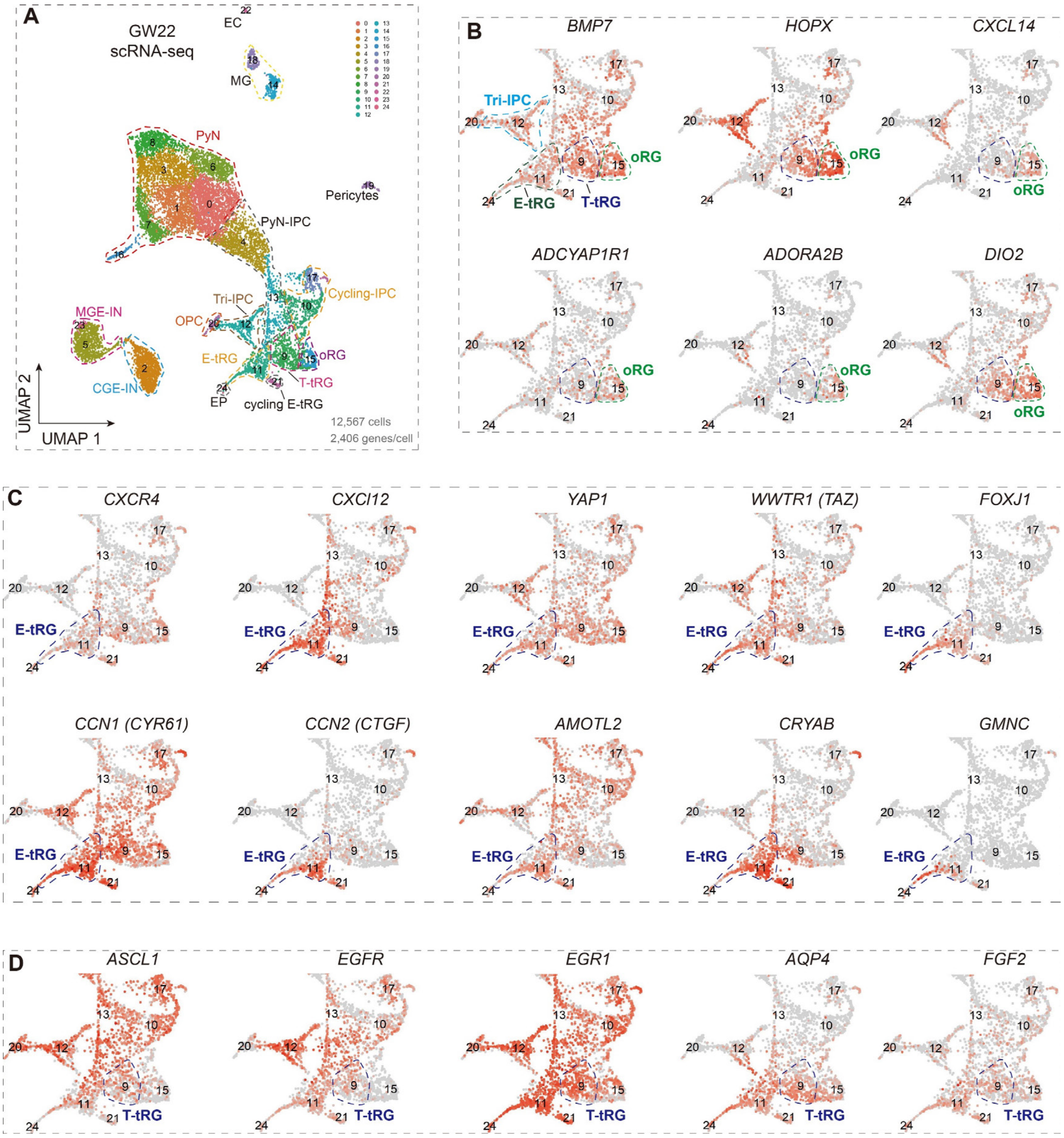

Figure S8. Expression of cell-type-specific markers in scRNA-Seq data at GW22. **A)** UMAP of GW22 scRNA-Seq cells colored by cluster (from Figure 1A). **B-D)** UMAP plots showing gene expression in human cortical progenitors. Note that key components of the cortical PKA signaling, including *ADCYAP1R1, ADORA2B, DIO2*, and *CXCL14*, demonstrate significantly elevated expression levels in oRGs (**B**). *FOXJ1* and *GMNC* (*GEMC1*) are two master regulators essential for initiating the multiciliation program. Note that the onset of FOXJ1 expression in E-tRGs precedes that of GMNC (**C**). oRG, outer radial glia; E-tRG, ependymocyte-generating truncated radial glia, T-tRG, Tri-IPC-generating tRG; EP, ependymal cell; Tri-IPCs, tripotential intermediate progenitor cells; OPC, oligodendrocyte-IPCs; PyN, cortical glutamatergic pyramidal neuron; PyN-IPC, PyN intermediate progenitor cells; CGE-IN, caudal ganglionic eminence interneuron; MGE-IN, medial ganglionic eminence interneuron; EC, endothelial cell; MG, microglia.

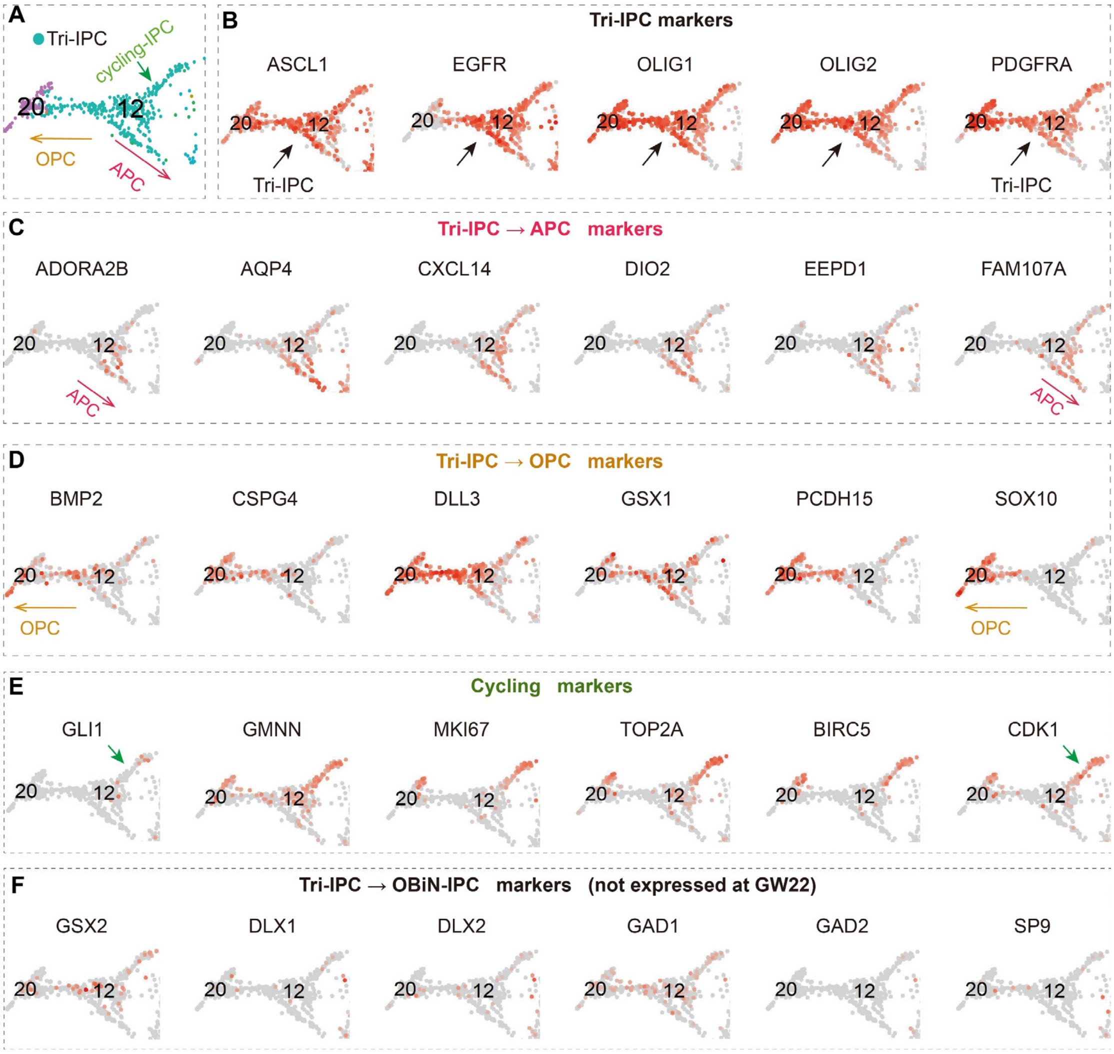

Figure S9. Human cortical T-tRG-derived Tri-IPCs at GW22 do not generate OBIN-IPCs. **A-E)** Marker genes for human cortical Tri-IPCs, APCs, OPCs, and cycling progenitors, are identified (arrows). **F)** OBIN-IPC marker genes are not observed in Tri-IPC clusters at GW22. APC, astrocyte-IPCs; OBIN-IPC, IPCs for cortically derived olfactory bulb interneuron.

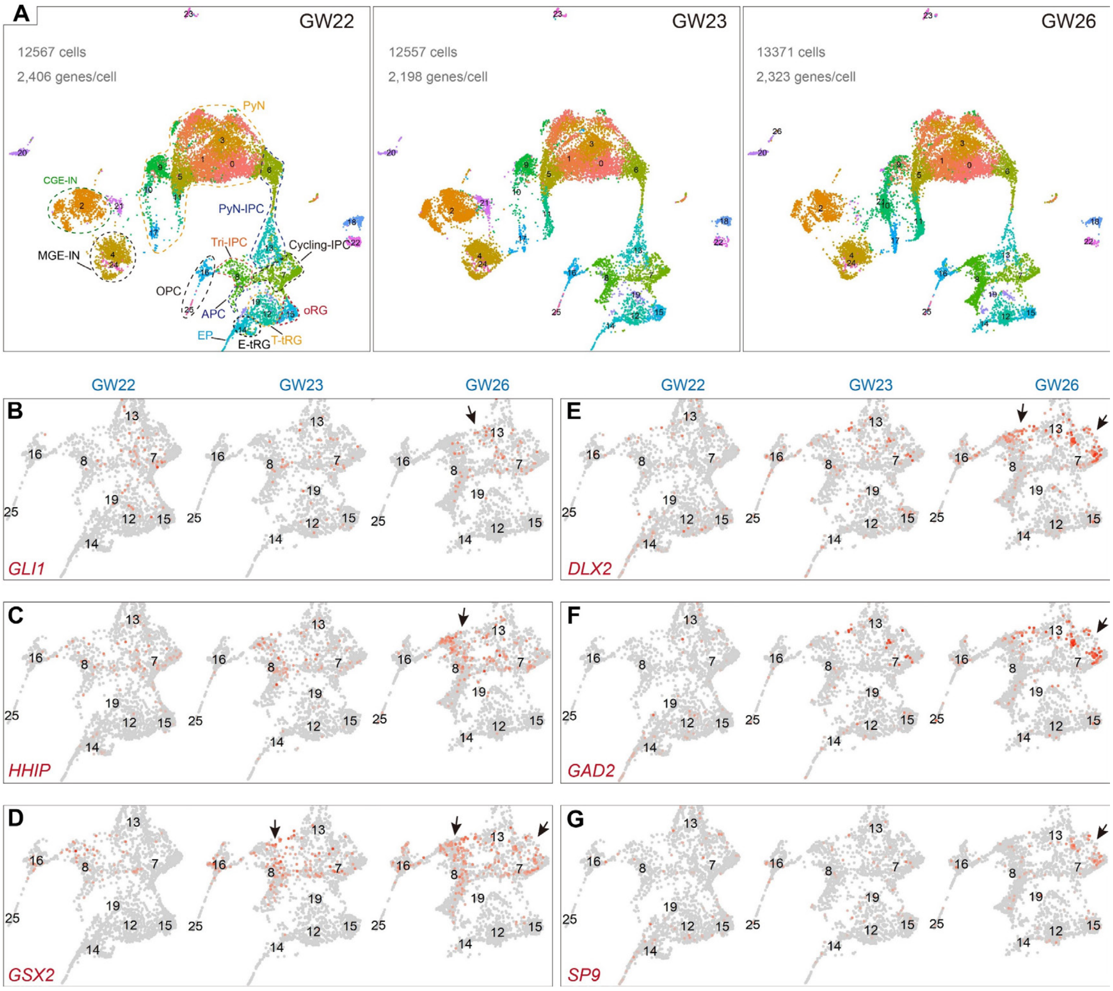

Figure S10. Human cortical T-tRG-derived Tri-IPCs at GW23 and GW26 undergo progressive generating OBIN-IPCs. A) UMAP of GW22, GW23, and GW26 scRNA-Seq cortical cells colored by cluster. **B-C)** Expression levels of SHH signaling markers *GLI1* and *HHIP* (arrows) gradually increase in human cortical progenitors from GW22 to GW26. **D-G)** Expression of OBIN-IPC markers, including *GSX2, DLX2, GAD2*, and *SP9* within Tri-IPC cluster (arrows), are observed at GW26.

Table S1. Antibodies used in this study

primary Ab	Species	Dilution	Manufacturer	Cat. No.
CUX1	Rabbit	1:200	Santa Cruz	sc-13024
CRYAB	Mouse	1:500	Abcam	ab13496
EGFR	Goat	1:1000	R&D System	BAF1280
EOMES	Guinea pig	1:500	Oasis Biofarm	OB-PGP022
FOXJ1	Mouse	1:1000	Invitrogen	14-9965-80
GFP	Chicken	1:3000	Aves labs	GFP-1020
GFAP	Rabbit	1:1000	Dako	Z0334
GSX2	Rabbit	1:500	Millipore	ABN162
NEUN	Rabbit	1:1000	Biosensis	R-3770-100
OLIG2	Rabbit	1:500	Millipore	AB9610
OLIG2	Rat	1:500	Oasis Biofarm	OB-PRB009
pERK1/2	Rabbit	1:400	Cell Signaling	#4370
SOX9	Rabbit	1:1000	Abcam	ab185966
YAP/TAZ	Rabbit	1:1500	Cell Signaling	8418S

Table S2. Single-cell RNA sequencing (scRNA-Seq) was performed on 11 mouse cortical samples newly generated for this study

Number	Age	Genotype	Notes
1	E15.0	*Map2k1/2* (without *Cre*)	Whole cortex
2	E15.0	*Emx1-Cre; Map2k1/2-dcko*	Whole cortex
3	E16.5	*Wild-type littermate*	FlashTag labeling at E15.5
4	E16.5	*hGFAP-Cre; SuperHippo*	FlashTag labeling at E15.5
5	E16.5	*SuperHippo* (without *Cre*)	FlashTag labeling at E15.5
6	E16.5	*Emx1-Cre; SuperHippo*	FlashTag labeling at E15.5
7	E18.0	*hGFAP-Cre;* $Smo^{F/F}$ + *dnPKA*	IUE (*dnPKA*) labeling at E15.0
8	P2	*SuperHippo* (without *Cre*)	FlashTag labeling at P0
9	P2	*hGFAP-Cre; SuperHippo*	FlashTag labeling at P0
10	P2	$Smo^{F/F}$	FlashTag labeling at P0
11	P2	*hGFAP-Cre;* $Smo^{F/F}$	FlashTag labeling at P0
